# Synthesis, Molecular Docking, and Biological Studies of New Naphthalene‐1,4‐Dione‐Linked Phenylpiperazine and Thioether

**DOI:** 10.1002/cbdv.202500774

**Published:** 2025-07-26

**Authors:** Berrin Yilmaz, Nahide Gulsah Deniz, Cigdem Sayil, Ozlem Bingol Ozakpinar, Merve Gurboga, Turgut Sekerler, Pervin Rayaman, Erkan Rayaman, Elif Caliskan Salihi

**Affiliations:** ^1^ Faculty of Engineering, Department of Chemistry, Division of Organic Chemistry Istanbul University‐Cerrahpaşa Istanbul Turkey; ^2^ Faculty of Pharmacy, Department of Biochemistry Marmara University, Basibuyuk Campus Istanbul Turkey; ^3^ Institute of Health Sciences, Department of Biochemistry (Pharmacy) Marmara University, Basibuyuk Campus Istanbul Turkey; ^4^ Faculty of Pharmacy, Department of Pharmaceutical Microbiology Marmara University, Basibuyuk Campus Istanbul Turkey; ^5^ Faculty of Pharmacy, Department of Basic Pharmaceutical Sciences Marmara University, Basibuyuk Campus Istanbul Turkey

**Keywords:** anticancer activity, antimicrobial activity, molecular docking, naphthoquinone, spectroscopy

## Abstract

Quinones are an important family of chemical compounds that are used in the development of anticancer drugs. Due to the anticancer properties of quinones, this study aims to investigate the antiproliferative and antimicrobial activities of newly substituted quinone derivatives synthesized through reactions with various nucleophiles (such as thiols and amines). The antiproliferative effects of the synthesized compounds on human cancer cell lines were screened at a concentration of 10 µM using the 3‐(4,5‐dimethylthiazol‐2‐yl)‐2,5‐diphenyltetrazolium bromide (MTT) assay. The cytotoxic effects of the synthesized compounds were also assessed in NIH/3T3 cells. Target molecules for the cancer cell lines A549, PC‐3, and MCF‐7 employed in this study were identified in light of the literature in order to perform molecular docking studies based on the MTT experiment results. Moreover, the antimicrobial effects of newly synthesized quinone derivatives were also investigated. Among the synthesized compounds, especially compound **10** had strong antiproliferative effects on a variety of cancer cell lines, most notably PC‐3, while having no cytotoxicity toward NIH/3T3 cells. Furthermore, compounds **5** and **8** exhibited antimicrobial activity against both bacteria and fungi. Our results indicate that the newly synthesized quinone molecules may function as therapeutic agents for the treatment of cancer and infectious diseases.

## Graphical abstract

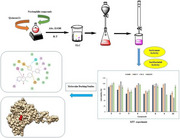

## 1 Introduction

Cancer is one of the most prevalent and lethal diseases worldwide, contributing to a significant number of deaths annually. Because current cancer therapy regimens lack selectivity and have many adverse effects, research into alternative treatment techniques is still important. For decades, scientists have continued to investigate potential new drug candidates for cancer treatment by exploring both naturally derived compounds and synthetic pharmaceutical agents produced in laboratory settings [1, 2].

Compounds with a naphthoquinone (NQ) framework are known to show strong activity against human breast, liver, cervical, and gastric cancer cells by inducing apoptosis [3, 4, 5]. The primary mechanism underlying the anticancer effect of quinones is thought to be the triggering of the autophagy system [6, 7, 8, 9] and the formation of the reactive oxygen species (ROS) [10, 11, 12], such as hydroxyl radicals [13]. Ansamycin antibiotics, substituted‐1,4‐NQ derivatives containing amino groups, represent a significant part of antibiotics with anticancer activity [14, 15]. Many NQ compounds are toxic to various cancer cell lines due to properties of redox potential [16, 17, 18, 19]. For example, it has been shown that some alkylamino quinone derivatives have a high cytotoxic effect against human colon, brain, and pancreatic cancer cells [20]. Ourworking group has investigated a variety of quinone compounds and their uses in anticancer research on quinone derivatives replaced with heterogroups [21, 22, 23, 24, 25, 26]. Furthermore, we discovered that 2‐(*tert*‐butylthio)‐3‐chloronaphthalene‐1,4‐dione, one of the NQ derivatives with a sulfur atom substitution, had a strong antiproliferative effect on HeLa cells with an IC50 value of 10.16 µM [27, 28, 29].

We planned to direct our synthesis toward the discovery of novel *N*‐, *S*‐substituted quinone analogues and investigate their antiproliferative activities against human lung, breast, and prostate cancer cells and their cytotoxic activities on mouse embryonic fibroblast cells in this study. The molecular docking approach is a simulation technique used to predict potential interactions in drug development processes. This method allows for the determination of interactions and binding affinities between drug candidate molecules and their target receptors. Drug development efforts can benefit from generating a prediction profile by analyzing the interactions between disease‐related macromolecules and potential therapeutic compounds, providing valuable insights to complement experimental studies. Given the anticancer properties of 1,4‐NQ derivatives, molecular docking studies were conducted to explore the interactions between newly synthesized naphthalene‐1,4‐dione‐linked phenylpiperazine and thioether derivatives and various cancer‐related targets in this study. Moreover, the antimicrobial activity potential of quinone derivatives was determined by the agar well diffusion method. As well, the minimal inhibitory concentrations (MICs) of quinone derivatives were determined.

## 2 Results and Discussion

### 2.1 Chemistry of 1,4‐NQ Derivatives

**SCHEME 1:**
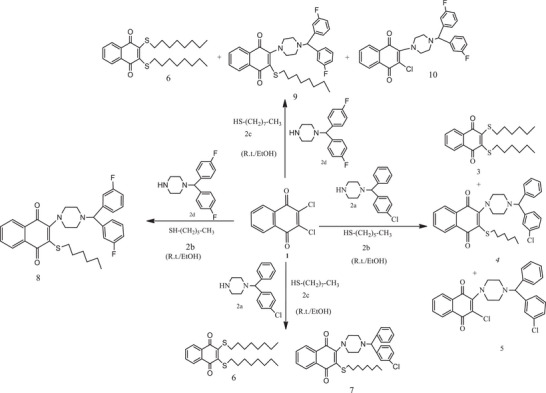
The synthesis of 1,4‐NQs (**3**–**10**).

The reaction of 2,3‐dihalo(Cl)‐1,4‐NQ (**1**) with some nucleophiles (**2a–d**) gave *S,S‐*, *N*‐, and *N,S*‐substituted quinone analogues (Scheme 1). Both halogen atoms, such as chlorine and bromine, can be replaced by using nitrogen‐containing heterocycles [30]. The one strong nucleophile property reacts with 2,3‐dihalo‐1,4‐NQ; only one chlorine atom can be displaced due to the electronic density of the quinone system. The displacement of the second chlorine atom and its separation from the structure can be easily achieved if the quinone system contains an electron‐withdrawing group (EWG) or in the presence of ethanol [31, 32, 33, 34, 35].

The synthesis of 1,4‐NQ derivatives (**3–10**) was investigated by the reactions of 2,3‐dihalo‐1,4‐NQ **(1)** with 1‐(4‐chlorobenzhydryl)piperazine (**2a**), hexanethiol (**2b**), octanethiol (**2c**), and 1‐[bis(4‐fluorophenyl)‐methyl]piperazine (**2d**) (Scheme 1).

In a previous study, heteroatom‐substituted NQ derivatives containing piperonylpiperazine or diphenylmethylpiperazine in Position two and chlorine atom in Position three of the 1,4‐NQ moiety showed good antiproliferative profiles and also antibacterial and antioxidant capacity. In the literature [31], the compounds of 2‐(*N*‐diphenylmethylpiperazin‐1‐yl)‐3‐chloro‐1,4‐NQ and 2‐[1‐piperonylpiperazin‐1‐yl]‐3‐chloro‐1,4‐NQ showed a strong cytotoxic effect on MCF‐7 cell lines and significant antibacterial activity against *Micrococcus luteum*. Interestingly, they also have powerful antioxidant capacity [31]. In this study, a similar compound, 2‐(4‐(bis(3‐fluorophenyl)methyl)piperazin‐1‐yl)‐3‐chloronaphthalene‐1,4‐dione, also gave similar results. The strong antimicrobial activity demonstrated by our similar compounds has encouraged us to further our studies on this subject.

### 2.2 Spectral Studies of Synthesized Compounds

As illustrated in Scheme 1, when starting compound (**1**) reacted in a single reaction vessel with an equimolar amount of various amines and thiols (**2a–d**). The products (**4** and **7**–**9**) were synthesized in good yields with appropriate reaction times and high purity. In the FT‐IR spectra of newly synthesized compounds, the stretching bands for the carbonyl group gave peaks in the range of 1537–1730 cm^−1^.

According to ^13^C(APT) NMR spectrum, except for synthesized quinone compounds (**4**, **5**, and **7–10**), two carbonyl signals were given between 178.05 and 182.90 ppm. It was proved that two different substituents were connected to the quinone unit. However, the individual carbonyl carbons (C═O) of compounds **3** and **6** provided single chemical shift values at *δ* 178.94 and 178.98 ppm, respectively. These ^13^C(APT) shift values have agreed well with the related literature [36, 37, 38, 39].

The respective molecular ion peaks were observed at *m*/*z* (%) *m*/*z* = 391.28329 [M]^+^ (compound **3**), 559.21674 [M + H]^+^ (compound **4**), 477.11203 [M]^+^ (compound **5**), 447.23743 [M]^+^ (compound **6**), 587.24829 [M]^+^ (compound **7**), 561.23718 [M]^+^ (compound **8**), 589.26807 [M]^+^ (compound **9**), and 479.13260 [M + H]^+^ (compound **10**), respectively, for the synthesized compounds (**3**–**10)** by using the ESI method of high‐resolution mass spectrum (HRMS) method.

### 2.3 Antiproliferative Activity of Synthesized Compounds

**FIGURE 1:**
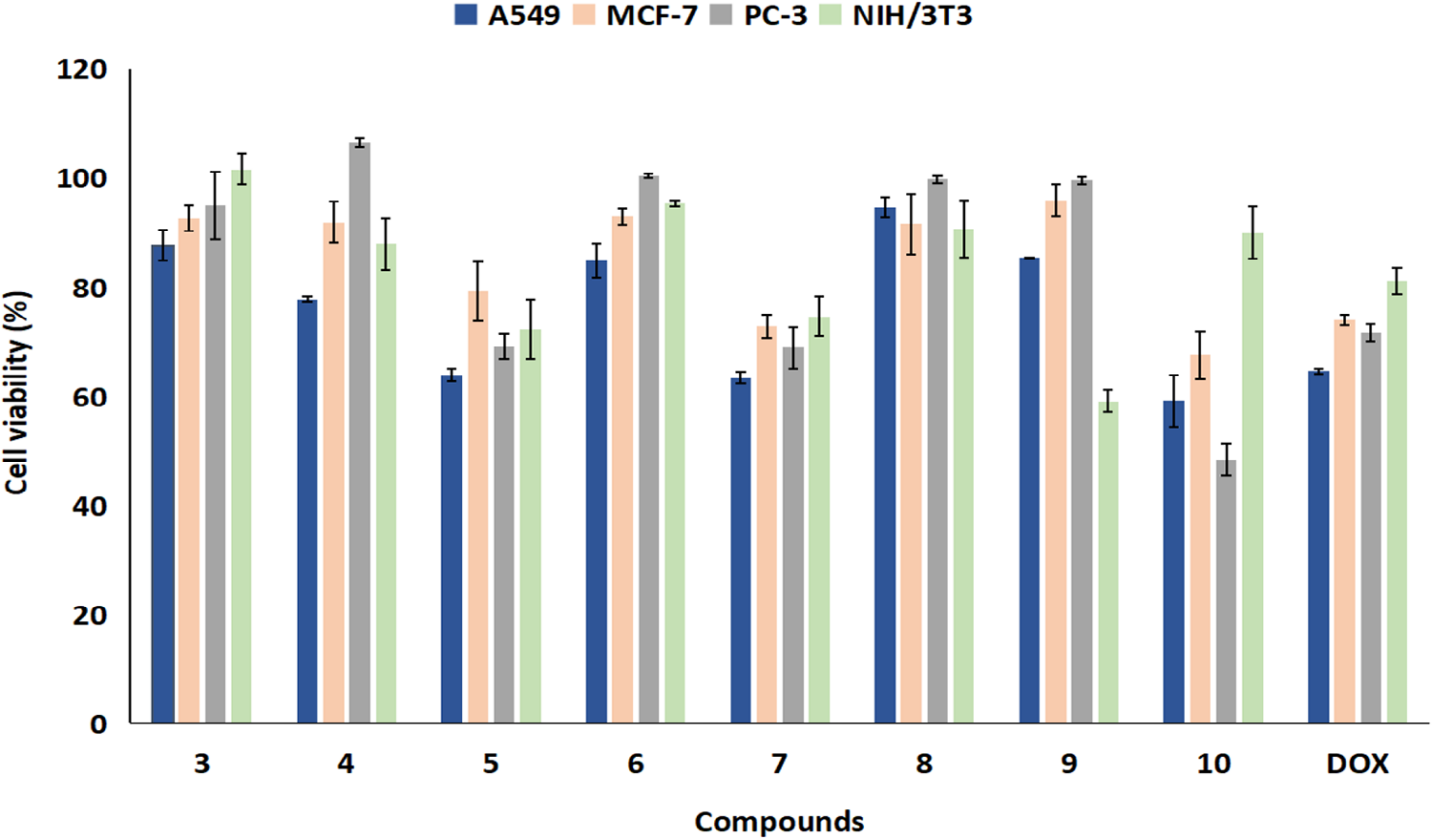
The effect of compounds on the viability of cancer cell lines and NIH/3T3 cells (*n* = 3). DOX, doxorubicin.

The antiproliferative effect of the synthesized compounds (**3**–**10**) on human cancer cell lines was screened at a concentration of 10 µM using the 3‐(4,5‐dimethylthiazol‐2‐yl)‐2,5‐diphenyltetrazolium bromide (MTT) assay. The effect of the compounds on the cell viability at 24 h is shown in Figure 1. The cytotoxic effects of the synthesized compounds were also assessed in NIH/3T3 cells. According to the results, compound **10** was determined to be the most effective compound against all cancer cell lines in this study. The highest inhibitory activity of compound *2‐(4‐(Bis(3‐Fluorophenyl)Methyl)Piperazin‐1‐yl)‐3‐Chloronaphthalene‐1,4‐Dion)* **10** was observed in PC‐3 cells (51.66%). Additionally, compound **10** has low toxicity toward healthy cells (10.1%), indicating that it does not significantly harm NIH/3T3 cells. Interestingly, compound **10** showed a stronger inhibition on cancer cell proliferation than the reference drug doxorubicin (DOX), which contains a 1,4‐NQ ring. On the other hand, the compounds **5** and **7** showed promising results against A549 cells (36.17% and 36.71%, respectively); however, the remaining compounds at 10 µM concentration had virtually no antiproliferative effects.

The fact that compound **10** has an effect on all three cancer cell lines is due to the fluorophenyl piperazine ring it contains. The biological effects of piperazine‐conjugated molecules might vary greatly, with the main one being anticancer action [40, 41]. Consistent with our results, a very recent study by Zsoldos et al. reported that 1‐bis(4‐fluorophenyl)methylpiperazine derivatives exhibited potent anticarcinogenic activity on all cancer cell lines used in the study [42].

### 2.4 Molecular Docking Studies of Compounds (**5**, **7**, and **10**)

On the basis of the results of the MTT experiment, docking was carried out among the three compounds (**5**, **7**, and **10**) that gave the highest cytotoxic activity in all three cancer cell lines and the target proteins. The selection of target proteins for molecular docking studies was based on their biological roles in cancer signaling pathways as supported by the literature [43, 44, 45]. The cell lines utilized in this study (A549, PC‐3, and MCF‐7) serve as model systems for lung, prostate, and breast cancers, respectively. For these cell lines, proteins such as epidermal growth factor receptor (EGFR), vascular endothelial growth factor receptor 2 (VEGFR2), mammalian target of rapamycin (mTOR), Caspase‐3, focal adhesion kinase (FAK), and cyclin‐dependent kinase 6 (CDK6) were selected due to their critical roles in the biology of the respective cancer types, as highlighted in previous studies. Comparison with docetaxel, which is used as a positive control, reveals that three synthesized quinone compounds (**5**, **7**, and **10**) exhibit significantly higher binding scores, as depicted in Table 2.

Simultaneously with the cytotoxic activity results, we observed that the molecule coded as compound **10** demonstrates excellent docking scores against all proteins. Furthermore, the highest score (−9.8 kcal/mol), as indicated in Table 1, is obtained from the docking experiment of the compound **10** molecule with the EGFR protein.

**TABLE 1:** Docking scores of compounds (**5**, **7**, and **10**) with proteins (kcal/mol).

Proteins	Docetaxel	5	7	10
EGFR	−8.5	−9.4	−8.9	−9.8
Caspase‐3	−7.1	−7.6	−6.4	−8
VEGFR2	−6.7	−8.4	−9	−9
mTOR	−8	−8.6	−8.7	−9.3
FAK	−6.6	−9.4	−8.7	−9.5
CDK6	−7.1	−9.8	−8.3	−9.3

Abbreviations: CDK6, cyclin‐dependent kinase 6; EGFR, epidermal growth factor receptor; FAK, focal adhesion kinase; mTOR, mammalian target of rapamycin; VEGFR2, vascular endothelial growth factor receptor 2.

The overexpression or mutation of EGFR is often associated with the poor prognosis, rapid metastasis, short‐term recurrence, and short survival time of epithelium tumors, such as breast, gastrointestinal, ovarian, prostate, and cervical cancer [46]. Increasing evidence has shown that EGFR inhibitors have great potential in cancer treatment, especially for non‐small cell lung cancer, colon cancer, and hepatocellular carcinoma, which has led to an increasing number of research studies based on the design and synthesis of EGFR inhibitors [43, 47]. NQs have been shown to be involved in various molecular processes that drive cancer cells to enter the apoptotic process, such as suppression of the EGFR–NF‐κB signaling pathway [48]. Furthermore, many studies have shown that 1,4‐NQ derivatives exhibit strong effects on various cancer cells as potential EGFR inhibitors [19, 29, 49].

Overexpression of EGFR activates pro‐oncogenic downstream signaling pathways, including PI3K/AKT/mTOR [50, 51]. This signaling plays key roles in oncogenesis‐related events, such as cell survival, cell proliferation, cell differentiation, and cellular apoptosis. In vitro studies have shown that PI3K/AKT/mTOR signaling plays an important role not only in the proliferation and apoptosis of prostate cancer cells [52] but also in migration and invasion [53]. In our study, compound **10** exhibits the strongest inhibitory effect on prostate cancer cells and shows a strong binding with mTOR, suggesting that this compound may be an alternative potential therapeutic agent for prostate cancer treatment by inhibiting PI3/AKT/mTOR signaling.

**FIGURE 2:**
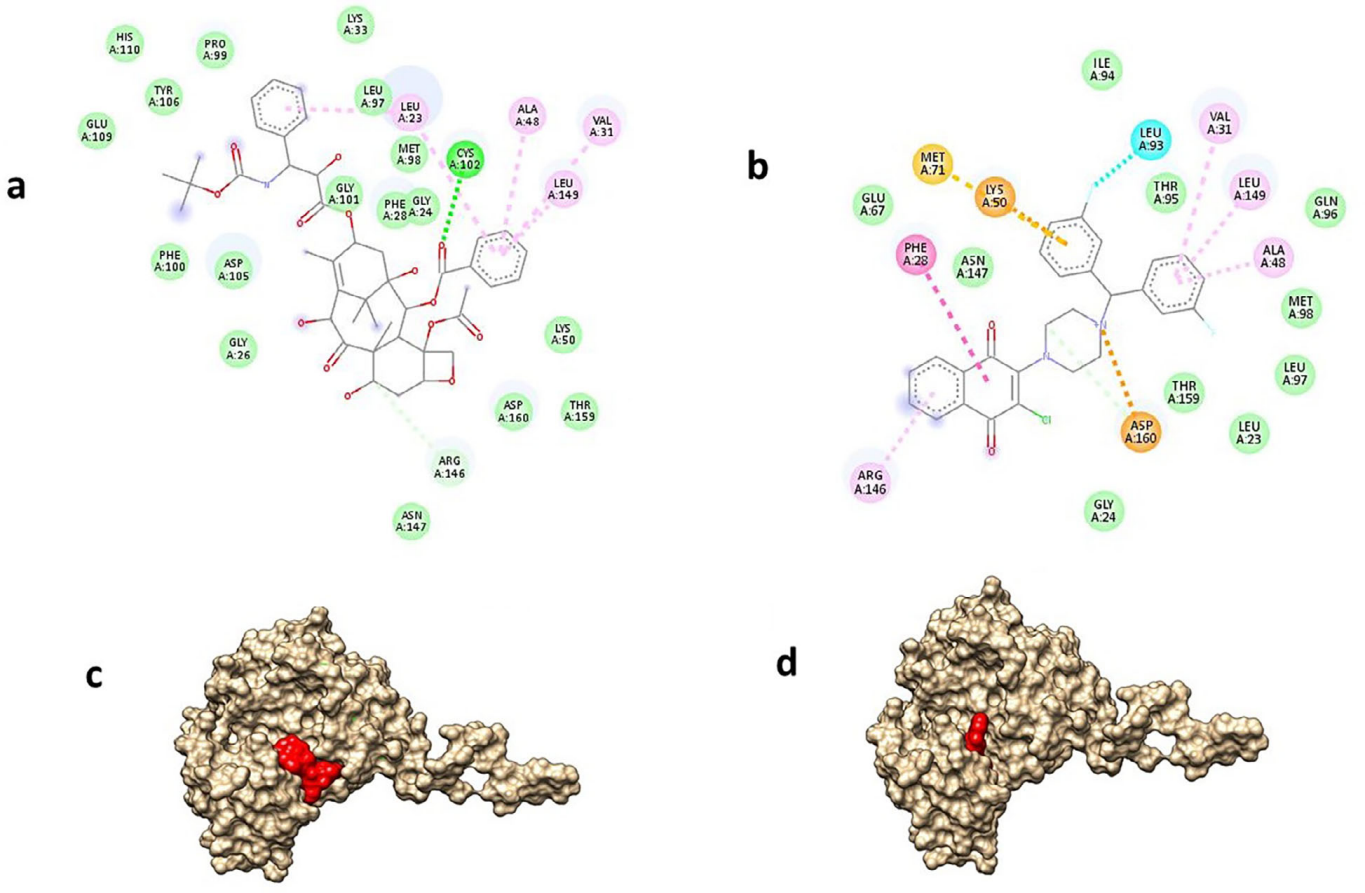
The interaction between docetaxel and EGFR (a) and docetaxel and compound **10** (b); 3D visualization of the molecular interaction between docetaxel and EGFR (c) and docetaxel and compound **10** (d).

Analyzing the protein–ligand interaction of the positive control and the compound **10** molecule when docked with EGFR, considering the numerical data, both molecules exhibit high‐density hydrophobic interactions with similar amino acids (Figure 2a,b). Moreover, it is noteworthy that both molecules position themselves in a comparable location within the active region of the protein (Figure 2c,d).

In conclusion, the docking studies were found to be in agreement with the in vitro antiproliferative data, particularly for compound **10**. The observed interactions between compound **10** and EGFR suggest a possible mechanism of action, which may help guide future experimental investigations.

### 2.5 Antimicrobial Activity of Quinone Derivatives

**FIGURE 3:**
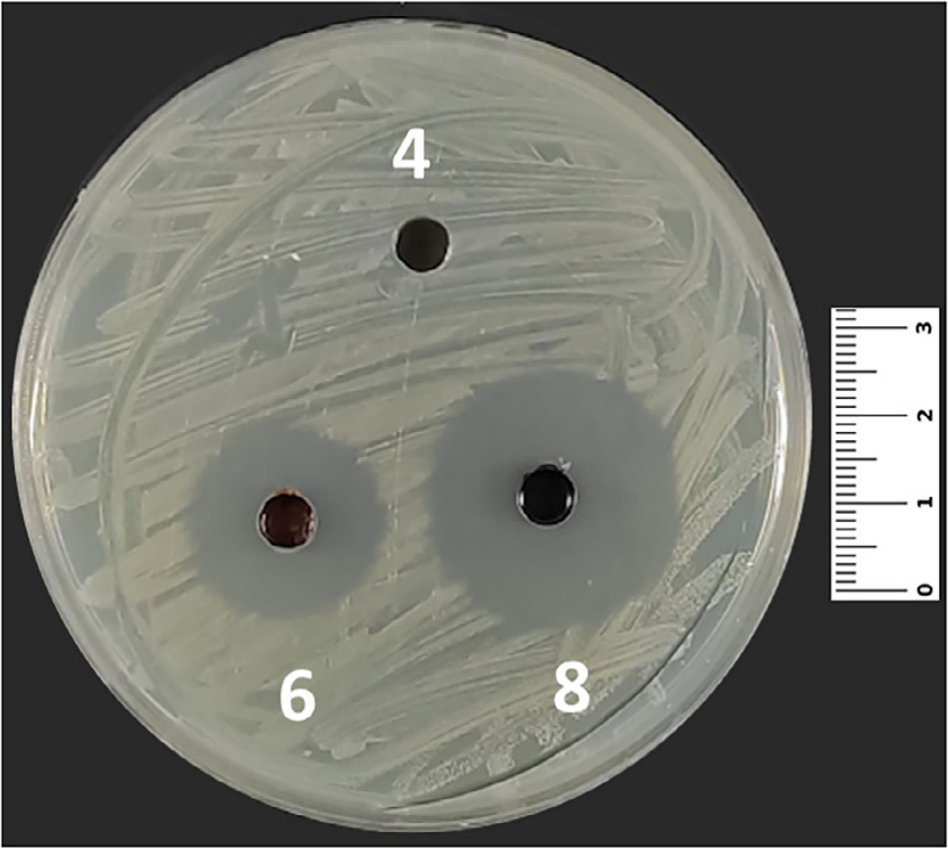
The antibacterial effect of compounds **4**, **6**, and **8** against *Staphylococcus aureus* ATCC 25923.

In the present study, compounds **5** and **8** were effective against all of the microorganisms, both bacteria and yeast. Compound **7** was effective against all of the microorganisms except *Escherichia coli* ATCC 25922, and compound **10**, a derivative, was effective against all of the microorganisms except *Staphylococcus aureus* ATCC 43300 (Table 3). Moreover, compound **3** was effective against *S. aureus* ATCC 25923 (Figure 3), *S. aureus* ATCC 43300, *Staphylococcus epidermidis* ATCC 11228, *Klebsiella pneumoniae* ATCC 4352, and *Candida albicans* ATCC 90028. Compounds **4** and **9** showed similar activities as being effective against *S. aureus* ATCC 43300, *S. epidermidis* ATCC 11228, *Pseudomonas aeruginosa* ATCC 27853, and *K. pneumoniae* ATCC 4352 (Table 2).

**TABLE 2:** Inhibition zones of quinone derivatives determined by the agar well diffusion method (mm).

Quinone derivatives	*Staphylococcus aureus* ATCC 25923	*Staphylococcus aureus* ATCC 43300	*Staphylococcus epidermidis* ATCC 12228	*Enterococcus faecalis* ATCC 29212	*Pseudomonas aeruginosa* ATCC 27853	*Escherichia coli* ATCC 25922	*Klebsiella pneumoniae* ATCC 4352	*Candida albicans* ATCC 90028
**3**	11.10 ± 0.18	27.87 ± 0.17	10.92 ± 0.19	0	0	0	9.46 ± 0.17	17.79 ± 0.24
**4**	0	17.45 ± 0.15	23.58 ± 0.22	0	15.46 ± 0.31	0	14.77 ± 0.27	0
**5**	15.13 ± 0.22	16.35 ± 0.24	15.73 ± 0.23	29.30 ± 0.21	12.22 ± 0.24	14.31 ± 0.17	11.93 ± 0.25	21.12 ± 0.11
**7**	21.00 ± 0.25	16.83 ± 0.23	16.70 ± 0.31	19.27 ± 0.20	13.18 ± 0.27	0	16.22 ± 0.25	16.37 ± 0.13
**8**	29.73 ± 0.11	15.28 ± 0.10	13.84 ± 0.14	12.96 ± 0.23	10.63 ± 0.10	11.41 ± 0.08	11.13 ± 0.08	17.40 ± 0.18
**9**	0	12.28 ± 0.10	15.42 ± 0.25	0	18.23 ± 0.21	0	19.41 ± 0.29	0
**10**	9.83 ± 0.14	0	14.13 ± 0.17	14.20 ± 0.15	7.32 ± 0.09	10.83 ± 0.21	12.67 ± 0.17	17.82 ± 0.31
**M (10 µg/mL)**	31.80 ± 0.29	29.68 ± 0.32	23.49 ± 0.42	18.44 ± 0.22	29.32 ± 0.21	30.12 ± 0.12	28.56 ± 0.25	—
**AmB (100 µg/mL)**	—	—	—	—	—	—	—	21.14 ± 0.27

*Note*: “—”: untested.

Abbreviations: AmB, Amphotericin B; M, meropenem.

**FIGURE 4:**
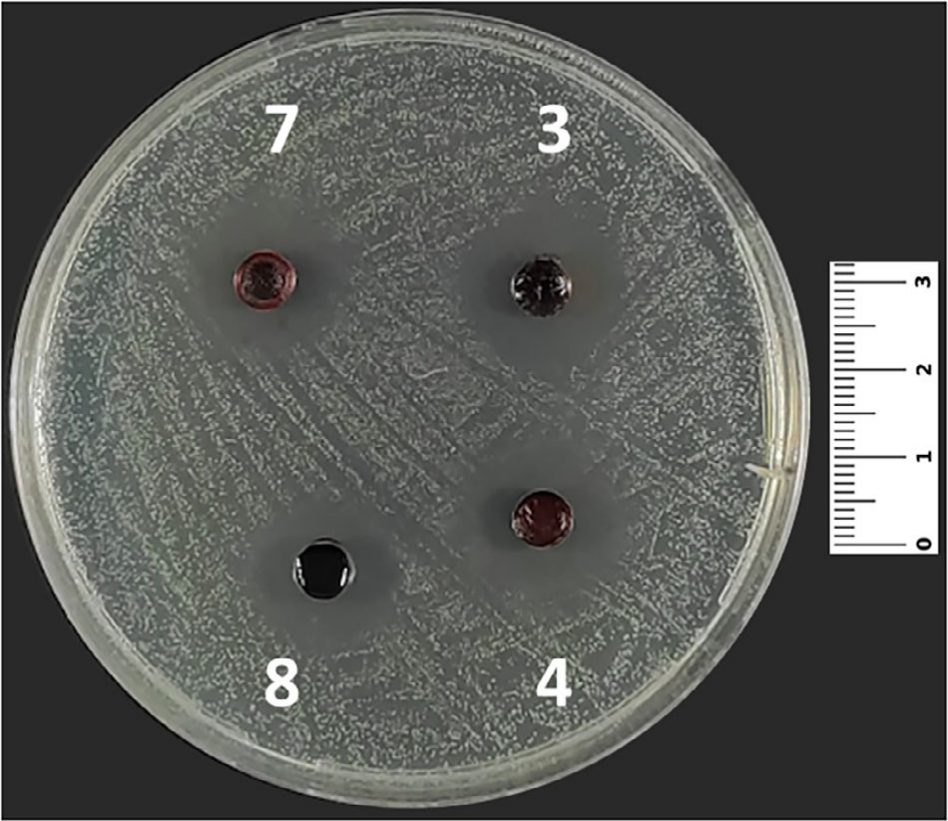
The antifungal effect of compounds **3**, **4**, **7**, and **8** against *Candida albicans* ATCC 90028.

Cho et al. investigated the antimicrobial effects of 1,4‐quinone derivatives against various microorganisms [54, 55, 56]. In their study, the authors declared that synthesized compounds **(3**–**5** and **7**–**10**) showed antibacterial effects against *S. aureus* and *P. aeruginosa*. Additionally, it was stated that the five synthesized compounds (**3**–**5**, **7**, and **9**) in our study have shown antifungal effects (Figure 4) against *C. albicans* at various concentrations of MIC (Table 3).

**TABLE 3:** The antimicrobial activity of quinone derivatives (**3**–**10**) (µM).

Compounds	*Staphylococcus aureus* ATCC 25923		*Staphylococcus aureus* ATCC 43300		*Staphylococcus epidermidis* ATCC 12228		*Enterococcus faecalis* ATCC 29212		*Pseudomonas aeruginosa* ATCC 27853		*Escherichia coli* ATCC 25922		*Klebsiella pneumoniae* ATCC 4352		*Candida albicans* ATCC 90028	
	MIC	MBC	MIC	MBC	MIC	MBC	MIC	MBC	MIC	MBC	MIC	MBC	MIC	MBC	MIC	MFC
**3**	3.51	6.99	112.01	224.01	224.01	448.03	>448.03	>448.03	>448.03	>448.03	>448.03	>448.03	56.02	112.01	112.01	224.01
**4**	>89.58	>89.58	11.20	22.40	44.79	89.58	>89.58	>89.58	11.20	22.40	>89.58	>89.58	22.40	44.79	>89.58	>89.58
**5**	1.63	3.27	26.18	52.36	52.36	104.72	26.18	52.36	6.56	13.09	26.18	52.36	6.56	13.09	26.18	52.36
**7**	3.73	7.46	14.90	29.80	14.90	29.80	29.80	59,60	7.46	14.90	>29.80	>29.80	14.90	29.80	14.90	29.80
**8**	64.65	129.30	16.16	32.33	64.65	129.30	64.65	129.30	32.33	64.65	32.33	64.65	8.08	16.16	16.16	32.33
**9**	>55.20	>55.20	6.90	13.81	13.81	27.60	>55.20	>55.20	27.60	55.20	>55.20	>55.20	13.81	27.60	>55.20	>55.20
**10**	36.60	73.20	>292.81	>292.81	73.20	146.40	146.40	292.81	146.40	292.81	73.20	146.40	18.30	36.60	146.40	292.81
**M**	0.0052	0.0104	0.0417	0.0834	0.0007	0.0013	0.0209	0.0417	0.0013	0.0052	0.0002	0.0003	0.0013	0.0052	—	—
**AmB**	—	—	—	—	—	—	—	—	—	—	—	—	—	—	1.08	4.33

*Note*: “—”: untested.

Abbreviations: AmB, Amphotericin B; M, meropenem; MBC, minimal bactericidal concentration; MFC, minimal fungicidal concentration; MIC, minimal inhibition concentration.

As it is known, quinone can be transformed to detrimental ROS by catalyzing redox cycles where NAP(P)H and molecular O_2_ take place. Moreover, some quinone derivatives have the ability to show their antiproliferative activity by impairing mitochondrial functions not only in bacteria (such as *S. aureus*) but also in fungi and protozoa [57].

In order to find out a solution for new agents against the antimicrobial resistance, Ulfah et al. have shown that diterpene quinone–derived compounds are safe to use. The investigators have stated that in vitro assay results of their study had shown that the antimicrobial activities of diterpene quinone–derived compounds were related to the inhibition of ATP as a function and disruption of the membrane function of *S. aureus*. Additionally, diterpene quinone–derived compounds exhibited good antibacterial activity. On the basis of in vitro and in silico assays, the mechanism of action of this compound is related to the disruption of bacterial membrane function, and it has the potential to inhibit the ATPase enzyme and the *S. aureus* gyrase [58].

In our opinion, the synthesized naphthalene‐1,4‐diones in our study (naphthalene‐1,4‐dione‐linked phenyl piperazine and thioether) might show efficacy against microorganisms by detrimental ROS by catalyzing redox cycles and impairing mitochondrial functions and bacterial membrane function and inhibiting the ATPase enzyme.

## 3 Conclusions

The new quinone analogues (**3–10**) were synthesized by the reaction of 1,4‐NQ (**1**) with heterocyclic ring‐containing nucleophiles such as thiols and piperazine derivatives in this study. The chemical structures of these newly synthesized compounds were elucidated by FT‐IR, ^1^H NMR, ^13^C(APT) NMR, and HRMS. In the past study, some synthetic NQ derivatives showed good antiproliferative profiles and also antibacterial and antioxidant capacity. The strong antimicrobial activity demonstrated by our similar compounds has encouraged us to further our studies on this subject.

The compounds were assessed for their ability to inhibit the growth of different cancer cell lines (MCF‐7, A549, PC‐3) and NIH/3T3 using the MTT assay. Compound **10** exhibited potent antiproliferative effects on various cancer cell lines, notably PC‐3, with minimal cytotoxicity toward NIH/3T3 cells. However, compounds **5** and **7** showed promising activity against A549 cells. These compounds offer the potential to treat cancer, and their different biological effects should be investigated in future studies.

Our molecular docking analysis revealed favorable binding affinities for compound **10**, particularly with EGFR, in alignment with its observed in vitro cytotoxicity. Although these findings are promising, they should be interpreted as preliminary insights that require further biological validation to confirm their relevance. These findings corroborate previous studies, which also employed docking methods and reported high‐affinity binding of quinone derivatives to EGFR. The potential interaction between quinone‐based compounds and EGFR has been previously explored in silico. For instance, Nafie et al. reported that quinoline‐based thiazolidinone derivatives exhibited significant binding affinity toward EGFR, suggesting their potential as anticancer agents. Similarly, Mohanty et al. conducted computational studies on quinazoline derivatives, highlighting their favorable interactions with the EGFR active site. These findings align with our docking results, further supporting the hypothesis that quinone derivatives may serve as effective EGFR inhibitors [59, 60]. Collectively, our findings indicate that quinone derivatives, especially compound **10**, may have potential as anticancer agents targeting EGFR; however, further in vitro and in vivo studies are necessary to validate this hypothesis. Further studies are warranted to validate these findings in vivo and explore the molecular mechanisms underlying compound **10**’s anticancer activity.

We have investigated the antimicrobial effects of various quinone derivatives, which we synthesized. The findings we have mentioned above are quite important, as the new quinone derivatives that we have synthesized have shown various antimicrobial effects against both important gram‐positive and gram‐negative bacteria, together with antifungal effect. Hence, these compounds show activity against various microorganisms that are human pathogens; considering this antimicrobial activity, our study may have potential for drug development. Accordingly, our study can be quite beneficial because it might shed light for future studies on this area.

## 4 Materials and Methods

### 4.1 Chemistry

Melting points were measured by using a Büchi B‐540 melting point apparatus. FT‐IR spectra (cm^−1^) were recorded as KBr pellets in Nujol mulls on a Shimadzu IR Prestige 21 model Diamond spectrometer (ATR method). ^1^H NMR and ^13^C(APT) NMR spectrums were obtained using a Varian Unity Inova (500 MHz) spectrometer by using TMS as the internal standard and deuterated chloroform as the solvent. HRMSs were obtained on an LC‐HRMS Thermo Q Exactive. The synthesized compounds were separated and purified by using column chromatography on silica gel (Fluka Silica gel 60, particle size 63–200 µm). Kieselgel 60 F‐254 plates were used for thin‐layer chromatography (TLC) All chemicals were of reagent grade and were used without further purification. Moisture was excluded from the glass apparatus with CaCl_2_ drying tubes.

### 4.2 General Method for the Synthesis of Compounds (**3**–**10**)

2,3‐Dichloro‐1,4‐NQ (**1**) was used as a starting compound, and nucleophiles were refluxed in abs. ethanol. The reaction mixture was monitored by TLC until the disappearance of starting material. The reaction mixture was extracted by using 30 mL of CHCl_3_, washed three times with water, and then dried using Na_2_SO_4_. Evaporator system was used to remove the extra amount of solvent. The crude dark residue was separated and purified by column chromatography and dried using a vacuum oven at the end [61, 62, 63].

The spectral charts are given in the Supporting Information section.

### 4.3 Anticancer Studies of NQ Compounds

#### 4.3.1 Cell Culture

Human lung cancer cell line A549 (ATCC, CCL‐185), human breast cancer cell line MCF‐7 (ATCC, HTB‐22), human prostate cancer cell line PC‐3 (ATCC, CRL‐1435), and mouse embryonic fibroblast cells NIH/3T3 (ATCC, CRL‐1658) were used in this study (Table 1). NIH/3T3, MCF‐7, and A549 cells were cultured in Dulbecco's Modified Eagle Medium/F12 (DMEM/F12), whereas PC‐3 cells were cultured in RPMI 1640 medium (Gibco, Rockville, MD, USA). All used media contain 10% v/v fetal bovine serum (FBS) and 1% v/v penicillin‐streptomycin (Gibco, Rockville, MD, USA). Cells were grown in a humidified incubator at 37°C with 5% CO_2_. In our study, DOX, which is one of the most well‐known antineoplastic drugs, was also used as a positive control in this method.

#### 4.3.2 Cell Viability Assay

The MTT assay (Vybrant MTT Cell Proliferation Assay Kit, Thermo Fisher Scientific) was utilized to investigate the antiproliferative and cytotoxic effects of the compounds [64]. The cells were seeded in a 96‐well plate (1 × 10^4^ cells per well) and incubated for 24 h. The next day, the cells were treated with a 10 µM concentration of compounds for 24 h. At the end of the period, MTT solution was added to each well at a final concentration of 0.5 mg/mL and incubated for an additional 4 h. Then, the purple formazan product was solubilized by adding 100 µL of SDS buffer. Absorption measurements were taken at wavelengths of 570 and 630 nm using a microplate reader (Biotech Instruments, Winooksi, VT, USA). Cell viability percentages were calculated with optical density.

### 4.4 Molecular Docking Studies of NQs

#### 4.4.1 Data, Database, and Tools

To conduct molecular docking studies for the cell lines A549, PC‐3, and MCF‐7 used in this study, target molecules were first determined in light of the literature. Accordingly, two protein targets were determined for each cell line (Table 4) [65, 66, 67]. Crystal structures of EGFR (EGFR tyrosine kinase), Caspase‐3, VEGFR2, mTOR, FAK, and CDK6 proteins were downloaded from the Protein Data Bank (PDB) (https://www.rcsb.org/). The PDB IDs are as follows, respectively: 1M17, 2XYG, 2OH4, 4JT6, 1MP8, and 3NUP.

**TABLE 4:** Target proteins identified for molecular docking.

Target protein	Cancer cell line
EGFR	A549
Caspase‐3	A549
VEGFR2	MCF‐7
mTOR	MCF‐7
FAK	PC‐3
CDK6	PC‐3

Abbreviations: CDK6, cyclin‐dependent kinase 6; EGFR, epidermal growth factor receptor; FAK, focal adhesion kinase; mTOR, mammalian target of rapamycin; VEGFR2, vascular endothelial growth factor receptor 2.

The structures of the ligands were obtained with Chimera software after converting the image files of the molecules into SMILES format. Proteins and ligands were pre‐prepared for molecular docking with USCF Chimera 1.14 software. Subsequently, docking studies were performed using AutoDock Vina 1.1.2 software to investigate molecular interactions. Biovia Discovery Studio Visualizer software was used for detailed examination and visualization of protein–ligand interactions. In the validation of our molecular docking processes, the Zhang Group web server was used to calculate the RMSD values (https://zhanggroup.org//DockRMSD/).

### 4.5 Molecular Docking Process

PDB was utilized to obtain the crystallographic structures of the proteins associated with cancer (lung, breast, and prostate). For the molecular docking study, the coordinates of the active sites of each protein are given in Table 5, based on the coordinates of the ligand. After obtaining SMILES formats of the ligands using 3D images, their 3D structures were obtained with the Build Structure module in Chimera 1.12 software. Then, the lowest energy conformations were obtained in mol2 format with the Docking Prepare module in Chimera software.

**TABLE 5:** Active site coordinates of proteins.

Proteins	PDB ID	*X*	*Y*	*Z*	Grid size
**EGFR**	1M17	21.54	−0.26	51.31	25 × 25 × 25
**Caspase‐3**	2XYG	35.8	37.75	32.75	20 × 20 × 20
**VEGFR2**	2OH4	2.98	33.97	16.03	26 × 26 × 26
**mTOR**	4JT6	49.51	1.48	−47.14	20 × 20 × 20
**FAK**	1MP8	35.55	−4.43	25.06	20 × 20 × 20
**CDK6**	3NUP	22.14	34.94	−7.51	20 × 20 × 20

Abbreviations: CDK6, cyclin‐dependent kinase 6; EGFR, epidermal growth factor receptor; FAK, focal adhesion kinase; mTOR, mammalian target of rapamycin; VEGFR2, vascular endothelial growth factor receptor 2.

Molecular docking energy calculations were made according to the standard protocol of AutoDock Vina 1.1.2 software. The best energy from 10 different bindings was obtained as a result of these processes and recorded in PDB format. Biovia Discovery Studio Visualizer software was used for more detailed protein–ligand interactions on the structure with the best binding energy.

### 4.6 Determination of Antimicrobial Activity of Quinone Derivatives

#### 4.6.1 Antimicrobial Activity Test

In order to find out the antimicrobial activity potential of quinone derivatives the agar well diffusion method was applied. Additionally, MICs were found for quinone derivatives. Later, moreover, the minimal bactericidal concentrations (MBCs) and minimal fungicidal concentrations (MFCs) of the derivatives were assessed.

#### 4.6.2 Agar Well Diffusion Test

In our study, *S. aureus* ATCC 25923, *S. aureus* ATCC 43300, *S. epidermidis* ATCC 11228, *Enterococcus faecalis* ATCC 29212, *P. aeruginosa* ATCC 27853, *E. coli* ATCC 25922, and *K. pneumoniae* ATCC 4352 were the bacteria, and also *C. albicans* ATCC 90028 was the yeast used. All of the bacteria were inoculated on the surface of Mueller–Hinton agar (MHA), left for incubation at 37°C for 24 h, and Sabouraud dextrose agar (SDA) was used for *C. albicans*, left for incubation at 35°C for 48 h. After that the formed colonies suspensions of bacteria were adjusted to 10^8^ cfu/mL and those of *C. albicans* to 10^6^ cfu/mL in accordance with McFarland 0.5 standard turbidity by using 0.85% NaCl physiological saline solution (PSS). The prepared bacterial suspensions were spread on the surface of the MHA by using sterile swabs and yeast suspension on the surface of SDA, and then by using sterile punch, a 5 mm diameter wells were opened on the surface of the media. Then 50 µL of the extracts dissolved in their solvents were put into the wells. While as a positive control for bacteria meropenem and for the yeast amphotericin were assessed, as negative controls solvent (DMSO) and PSS were appraised. After incubation of the bacteria‐seeded plates at 37°C for 18–24 h and yeast‐seeded ones at 35°C for 24–48 h, the formed inhibition zones were measured and expressed in mm. All of the experiments were done in a triplicate average range [56, 68, 69].

#### 4.6.3 Assessment of MIC for Bacteria

In our study, the assessment of MIC for bacteria was done in accordance with the standards of the Clinical and Laboratory Standards Institute (CLSI) [70]. Here, although meropenem was used as positive control, CAMHB and DMSO were used as negative control [70].

#### 4.6.4 Assessment of MIC for Yeasts

The assessment of MIC for yeast was done in accordance with the standards of the CLSI [71]. Here, although Amphotericin B was used as positive control, RPMI and DMSO were used as negative control [71].

#### 4.6.5 Assessment of MBC and MFC

In order to assess the MIC and MFC values of the quinone derivatives, inoculation to the appropriate plates was done from each well of the microplate. Then the plates were left to incubate at 37°C for 24 h for bacteria and 48 h for the yeast. Finally, MBC and MFC values were determined according to the lowest values where no growth was observed [72].

## Author Contributions

**Berrin Yilmaz**: methodology, software, formal analysis. **Nahide Gulsah Deniz**: conceptualization, investigation, supervision, writing – original draft. **Cigdem Sayil**: conceptualization, investigation, supervision, writing – original draft. **Ozlem Bingol Ozakpinar**: data curation, formal analysis, software. **Merve Gurboga**: data curation, formal analysis, software. **Turgut Sekerler**: formal analysis, software. **Pervin Rayaman**: data curation, formal analysis, software. **Erkan Rayaman**: formal analysis, software. **Elif Caliskan Salihi**: data curation, formal analysis, software, writing – review and editing, supervision.

## Conflicts of Interest

The authors declare no conflicts of interest.

## Supporting information




**Supporting File 1**: cbdv70293‐sup‐0001‐SuppMat.doc

## Data Availability

The data that support the findings of this study are available from the corresponding author upon reasonable request.
