# Supplementary material for: Synthesis, Molecular Docking, and Biological Studies of New Naphthalene‐1,4‐Dione‐Linked Phenylpiperazine and Thioether
Source: Chem Biodivers. 2025 Jul 26;22(11):e00774. doi: 10.1002/cbdv.202500774 (PMC12629165; doi:10.1002/cbdv.202500774)
Supplement: Supplementary file 1 — Supporting File 1: cbdv70293‐sup‐0001‐SuppMat.doc [file CBDV-22-e00774-s001.doc]

**Supporting Information**

*(2,3-Bis(Hexylthio)Naphthalene-1,4-Dione)* **(3),** *(2-(4-((3-Chlorophenyl)(Phenyl)Methyl Piperazin-1-yl)-3-(Octylthio)Naphthalene-1,4-Dione* **(4)** *and Chloro-3-(4-((3-Chlorophenyl (Phenyl)Methyl) Piperazin-1-yl)Naphthalene-1,4-dione)* **(5)**

According to procedure 1; 2,3-Dihalo-1,4-NQ **(1)** (2.0 g, 8 mmol) was reacted with 1-(4-Chlorobenzhydryl)-Piperazine **2a** (2.53 g, 11.1 mmol) and hexanethiol**2b** (1.04 g 8.7 mmol) in Ethanol (EtOH) with presence of Na_2_CO_3_ at room temperature. Compounds (2,3-bis(hexylthio)naphthalene-1,4-dione) **(3)** [60] and new (2-(4-((3-Chlorophenyl)(phenyl)methyl piperazin-1-yl)-3-(octylthio)naphthalene-1,4-dione) **(4)**, Chloro-3-(4-((3-Chlorophenyl(phenyl)methyl)piperazin-1-yl)naphthalene-1,4-dione) **(5)** were synthesized.

***Compound* (3)** [60]: Dark red solid, Yield: 0.926 g, 16%, R*_f_* : 0.93 [(2:1)CHCl_3_:PET)], Mp: 51.3-51.5℃. ^1^H-NMR (499.74 MHz, CDCl_3_): δ = 0.74-0.77 (t, 6H, 2CH_3_), 1.16-1.58 (m, 16H, CH_2_), 3.14-3.18 (t, 4H, 2S-CH_2_), 7.55-7.65 (m, 2H, CH_napht_), 7.90-7.94 (m, 2H, CH_napht._). ^13^C (APT) NMR (125.66 Hz, CDCl_3_) δ: 13.98 (CH_3_), 22.49-31.30 (-CH_2_), 34.96 (S-CH_2_), 76.81 (–CH<), 126.79, 126.98, 133.00, 133.35 (CH_arom_, C_arom_), 178.94 ppm (C=O) ppm, FTIR(ATR); 3021.45 (CH_arom_), 2921.63, 2953.45 (CH_alip_), 1502.28 cm^-1^ (C=C), 1650.77, 1587.13 cm^-1^ (C=O). C_22_H_30_O_2_S_2_ (M_w_=390.6 g/mol). HRMS: m/z=391.28329 (M)^+^.

**Compound (4):** Dark orange oil, Yield: 0.481 g, 8.64%, R*_f_*: 0.71, [(2:1) CHCl_3_:PET)], ^1^H-NMR (499.74 MHz, CDCl_3_): δ = 0.85-0.90 (t, 3H, CH_3_), 1.25-1.68 (m, 8H, CH_2_), 3.18-3.22 (t, 2H, S-CH_2_), 4.37-4.41 (q, 8H, CH_2piper_), 7.58-7.62 (m, 9H, CH_arom_), 7.94-8.00 (m, 4H, CH_napht._). ^13^C(APT)NMR (125.66 Hz, CDCl_3_) δ: 13.99 (CH_3_), 22.50-33.10 (CH_2_), 34.96 (S-CH_2_), 76.8 (–CH<), 126.79, 126.98, 133.00, 133.3, 132.35 (CH_arom_, C_arom_), 126.8 (=C-N), 178.94, 182.9 ppm (C=O). FTIR(ATR); 3067.23 cm^-1^(CH_arom_), 2854.13, 2924.52, 2954.41 cm^-1^ (CH_aliph_), 1540.28 cm^-1^ (C=C), 1650.77, 1587.13 cm^-1^(C=O). C_33_H_35_ClN_2_O_2_S (M_w_= 558.16 g/mol). HRMS: m/z= 559.21674 (M+1H)^+^

**Compound (5):** Dark red solid, Yield: 4.058 g, 72.85%, R*_f_*: 0.46, [(2:1) CHCl_3_:PET)], Mp.: 104.88-105.10℃.  ^1^H-NMR (499.74 MHz, CDCl_3_): δ = 2.47-2.51 (bs, 8H, CH_2piper_), 4.30(s,1H, –CH<), 7.14-7.34 (m, 9H, CH_arom_), 7.52-7.60 (m, 2H, CH_napht._), 7.86-7.90 (dt, 1H, CH_napht._), 7.96-7.98 (dt,1H, CH_napht._). ^13^C(APT)NMR (125.66 Hz, CDCl_3_) δ: 22.55-34.73 (N-CH_2_), 76.76 (–CH<), 125.85, 126.22, 127.33, 127.85, 132.18, 132.96 (CH_arom_, C_arom_), 110.0 ppm (-Cl-C_napht._), 129.21 (=C-N), 181.85, 182.06 ppm (C=O). FTIR(ATR); 2954.41, 2918.73 cm^-1^ (CH_aliph_), 3044.12 cm^-1^ (CH_arom_), 1665.23, 1537.27 cm^-1^ (C=O), 1523.49 cm^-1^ (C=C). C_27_H_22_Cl_2_N_2_O_2_ (M_w_=477.48 g/mol). HRMS: m/z=477.11203 (M)^+^.

*(2,3-Bis(Octylthio)Naphthalene-1,4-Dion)* **(6)** and*(2-(4-((3-Chlorophenyl)(phenyl) methyl)Piperazin-1-yl)-3-Octylthio-Naphthalene -1,4-Dion) compound* **(7)**

According to procedure 1; 2,3-Dihalo-1,4-NQ **(1)** (2.0 g, 8 mmol) was reacted with 1-(4-Chlorobenzhydryl)-Piperazine **2a** (2.53 g, 11.1 mmol) and octanthiol **2c** (1.28 g 8.8 mmol) in EtOH with presence of Na_2_CO_3_ at room temperature. Compounds (2,3-bis(octylthio)naphthalene-1,4-dione) **(6)** [61] and new (2-(4-((3-Chlorophenyl)(phenyl)methyl)Piperazine-1-yl)-3-octylthio-Naphthalene-1,4-Dion) **(7)** were synthesized.

**Compound (6)** [61]: Purple solid, Yield: 1.50 g, 25.53%, R*_f_*: 0.71, Mp: 59.8-60.1℃ [(2:1) CHCl_3_: PET], ^1^H-NMR (499.74 MHz, CDCl_3_): δ = 0.78-0.80 (t, 6H, 2CH_3_), 1.16-1.60 (m, 24H, CH_2_), 3.15-3.20 (t, 4H, 2S-CH_2_), 7.58-7.62 (m, 2H, CH_napht._), 7.94-7.98 (m, 2H, CH_napht._). ^13^C(APT)NMR (125.66 Hz, CDCl_3_) δ: 13.93 (CH_3_), 22.49-34.14 (C-CH_2_), 35.10 (S-CH_2_), 133.33, 133.57 (CH_arom_,C_arom_), 178.98 ppm (C=O). FTIR(ATR); 3042.17(CH_arom_), 2972.73, 2950.55 (CH_alip_), 1649.80, 1587.13 (C=O), 1502.28 cm^-1^ (C=C). C_26_H_38_O_2_S_2_ (M_w_=446.71 g/mol). HRMS: m/z=447.23743 (M)^+^.

**Compound (7):** Purple solid, Yield: 2.85 g, 48%, R*_f_*: 0.28, Mp: 77.9-78.2°C [(2:1) CHCl_3_]: PET), ^1^H-NMR (499.74 MHz, CDCl_3_): δ = 0.87-0.91 (t, 3H, CH_3_), 1.27-1.54 (m, 12H, CH_2_), 2.40-2.45 (bs,8H, CH_2piper_), 3.48-3.51 (t, 2H, S-CH_2_), 4.30 (s,1H, –CH<), 7.10-7.34 (m, 9H, CH_arom_), 7.50-7.58 (m,1H, CH_napht_), 7.85 (dd, 1H, CH_napht._), 7.92 (dd,2H, CH_napht._). ^13^C(APT)NMR (125.66 Hz, CDCl_3_) δ: 14.09 (CH_3_), 22.60-33.70(CH_2_), 34.01 (S-CH_2_), 52.66 (N-CH_2_),75.54(CH<), 125.8, 126.50, 128.71, 132.1, 132.7, 132.9(CH_arom_, C_arom_), 126.2(=CN), 181.84,182.05 (C=O) ppm. FTIR(ATR); 3062.45 cm^-1^ (CH_arom_), 2966.95, 2920.66 cm^-1^ (C-H), 1665.23, 1638.23 cm^-1^ (C=O), 1524.45 cm^-1^(C=C). C_35_H_39_ClN_2_O_2_S (M_w_ = 587.21 g/mol). HRMS: m/z = 587.24829 (M)^+^.

*(2-(4-(Bis(3-Fluorophenyl)Methyl)Piperazine-1-yl)-3-(Hexylthio)Naphthalene-1,4-Dione* **(8):**

According to procedure 1; 2,3-Dihalo-1,4-NQ **(1)** (2.0 g, 8 mmol) was reacted with 1-Bis(4-Fluorophenyl)-Methyl Piperazine **2d** (2.54 g, 11.1 mmol) and hexanethiol**2b** (1.04 g 8.8 mmol) in EtOH with presence of Na_2_CO_3_ at room temperature. Compound (2-(4-(Bis(3-Fluorophenyl)Methyl)Piperazine-1-yl)-3-(Hexylthio)Naphthalene-1,4-Dion**(8)** was synthesized.

**Compound (8):** Orange oil, Yield: 0.86 g, 15.41%, R*_f_*: 0.71 [(2:1) CHCl_3_: PET)], ^1^H-NMR (499.74 MHz, CDCl_3_): δ = 0.86-0.89 (t, 3H, CH_3_), 1.20-1.45 (m, 8H, CH_2_), 2.45-2.50 (bs,8H, CH_2piper_), 3.48-3.51 (t, 2H, S-CH_2_), 4.30 (s,1H, –CH<), 7.35-7.44 (m, 8H, CH_arom_), 7.61-7.68 (m, 1H, CH_napht_), 7.96-7.99 (m, 1H, CH_napht_), 8.04-8.07 (m,2H, CH_napht._). ^13^C(APT)NMR (125.66 Hz, CDCl_3_) δ: 14.02 (CH_3_), 22.55-31.33 (CH_2_), 34.72 (S-CH_2_), 76.77 (–CH<), 126.01, 126.23, 132.74, 133.56, 132.95, 129.32 (CH_arom_, C_arom_), 137.92 (=C-N), 181.82, 182.06 ppm (C=O). FTIR (ATR); 3054.44 cm^-1^ (CH_arom_), 2969.84, 2901.38 cm^-1^ (C-H_alif_), 1502.28 cm^-1^ (C=C), 1649.87, 1590.02 cm^-1^ (C=O). C_33_H_34_F_2_N_2_O_2_S (M_w_=560.70 g/mol). HRMS: m/z=561.23718 (M)^+^.

*(2,3-Bis(Octylthio)Naphthalene-1,4-Dion)* **(6),** *(2-(4-Bis(3-Fluorophenyl)Methyl) Piperazin-1-yl)-3-(Octylthio)Naphthalene-1,4-Dion)* **(9)** *and (2-(4-(Bis(3-Fluorophenyl) Methyl)Piperazin-1-yl)-3-Chloronaphthalene-1,4-Dion)* **(10)**

According to procedure 1; 2,3-Dihalo-1,4-NQ **(1)** (2.0 g, 8 mmol) was reacted with 1-Bis(4-Fluorophenyl)-Methyl Piperazine **2d** (2.54 g, 11.1 mmol) and octanethiol **2c** (1.28 g 8.8 mmol) in EtOH with presence of Na_2_CO_3_ at room temperature. Compounds (2,3-Bis(Octylthio)Naphthalene-1,4-Dion) **(6)** [61] and new (2-(4-Bis(3-Fluorophenyl)Methyl)Piperazine-1-yl)-3-(Octylthio)Naphthalene-1,4-Dion) **(9**), (2-(4-(Bis(3-Fluorophenyl)Methyl)Piperazine-1-yl)-3-Chloronaphthalene-1,4-Dion) **(10)** were synthesized.

**Compound (9)**: Dark red oil, Yield: 0.977 g, 18.50%, R*_f_*: 0.60 [(2:1) CHCl_3_:PET)], ^1^H-NMR (499.74 MHz, CDCl_3_): δ = 0.77-0.91 (t, 3H, CH_3_), 1.20-1.57 (m, 12H, CH_2_), 2.45-2.50 (bs,8H, CH_2piper_), 3.50-3.60 (t, 2H, S-CH_2_), 4.23 (s,1H, –CH<), 7.35-7.45 (m, 8H, CH_arom_), 7.58-7.71 (m, 1H, CH_napht._), 7.88-7.92 (m, 2H, C_napht._), 7.96-8.00 (m,1H, CH_napht._). ^13^C(APT)NMR (125.66 Hz, CDCl_3_) δ: 14.09 (CH_3_), 22.64-31.78 (CH_2piper_), 34.70 (S-CH_2_), 77.03 (–CH<), 126.01, 126.23, 125.51, 133.04, 132.95, (CH_arom_, C_arom_), 129.31 (=C-N), 181.80, 182.03 ppm (C=O). FTIR(ATR); 3054.44 (CH_arom_), 2951.52, 2918.75 cm^-1^ (CH_alif_), 1649.87, 1588.02 cm^-1^(C=O), 1502.22 cm^-1^ (C= C). C_35_H_38_F_2_N_2_O_2_S (M_w_= 588.75 g/mol). HRMS: m/z=589.26807 (M)^+^

**Compound (10):** Dark red solid, Yield: 2.406 g, 41.28%, R*_f_* : 0.42 [(2:1) CHCl_3_:PET)], Mp: 96.6-96.8°C. ^1^H-NMR (499.74 MHz, CDCl_3_): δ = 2.45-2.50 (bs, 8H, CH_2piper_), 4.21 (s,1H, –CH<), 6.90-7.60 (m, 1H, CH_arom_), 7.90-7.92 (dd, 2H, CH_napht_), 8.21-8.24 (dd,2H, CH_napht._). ^13^C(APT)NMR (125.66 MHz, CDCl_3_): δ = 76.76 (–CH<), 110.00, 126.58, 126.89, 128.20, 129.24, 129.92, 133.07 (CH_arom_, C_arom_), 160.95,162.92 (-F-C_arom_),178.05, 181.88 (C=O). FTIR(ATR); 3022.58 cm^-1^ (CH_arom_), 2954.41, 2915.84 cm^-1^ (CH_alip_), 1730.80, 1679.69 cm^-1^ (C=O), 1459.85 cm^-1^(C=C), HRMS C_27_H_21_ClF_2_N_2_O_2_ (M_w_= 478.13 g/mol). MS: m/z = 479.13260 (M+H)^+^.

**
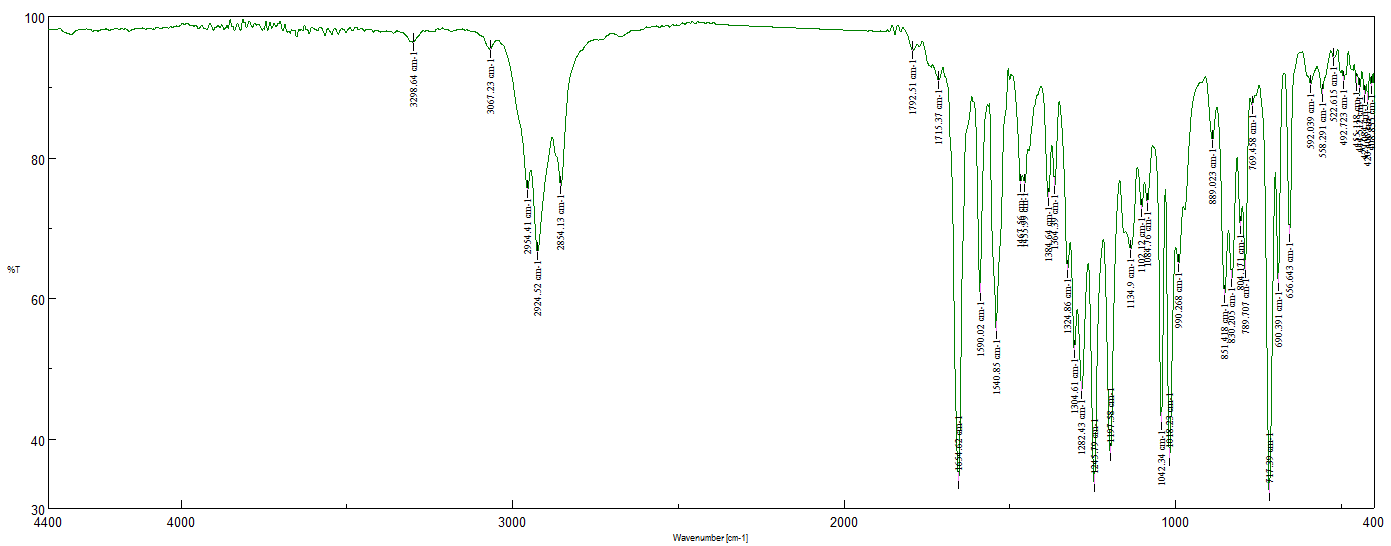
**

**S1.** FTIR Spectra of Compound **3**


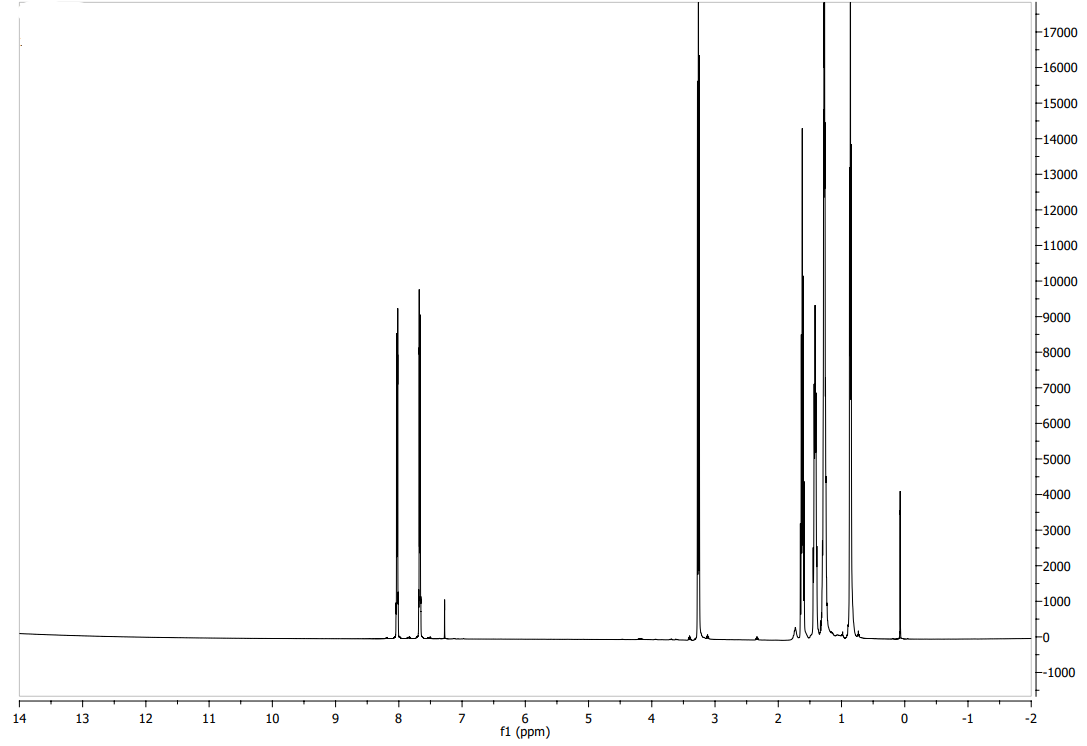


**S2**. ^1^H NMR Spectrum of Compound **3**


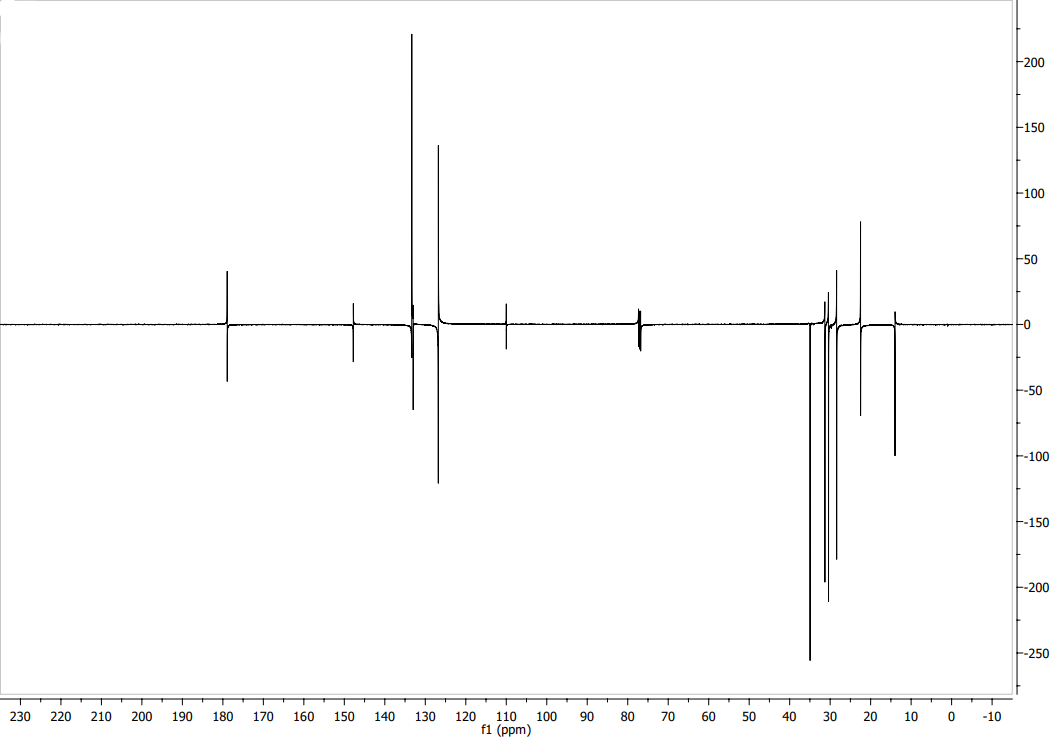


**S3.** ^13^C NMR(APT) Spectrum of Compound **3**

**
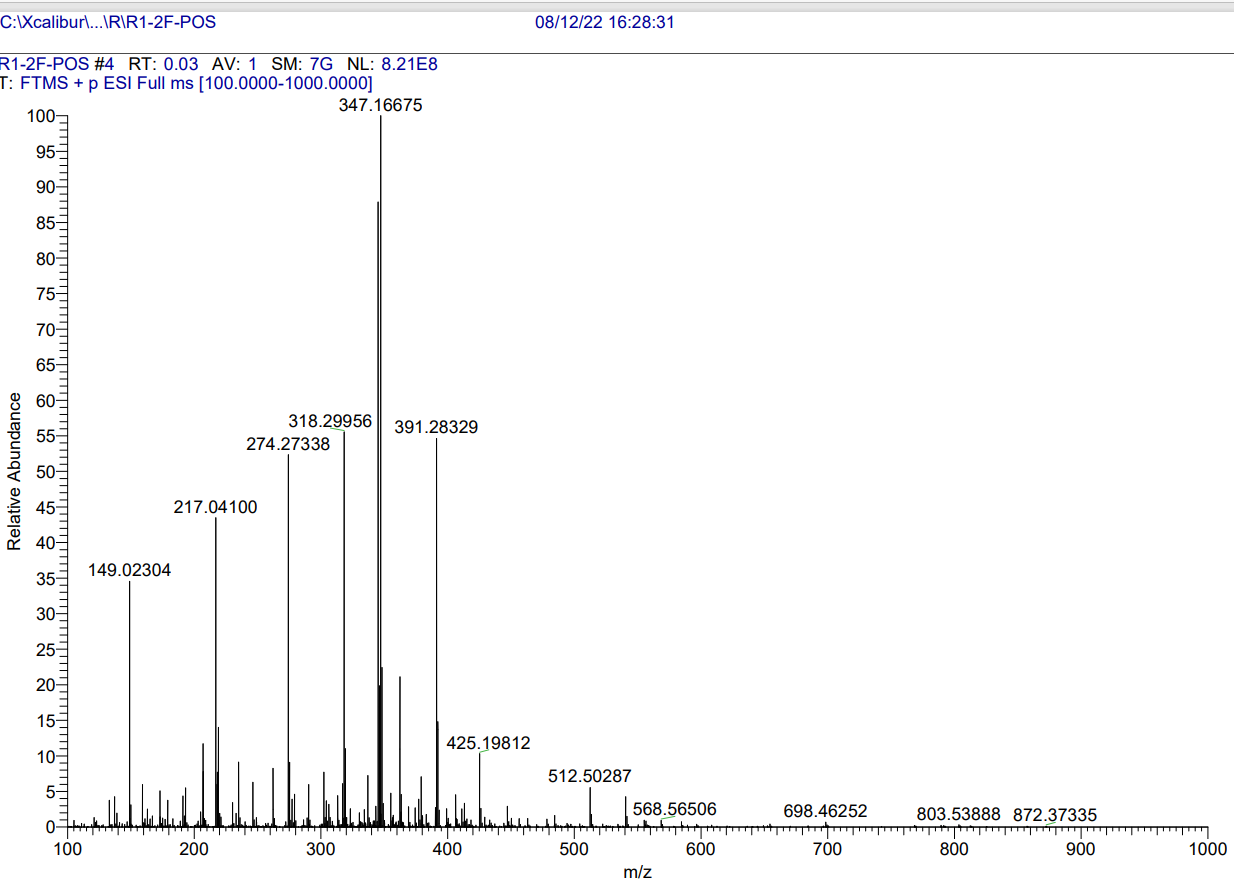
**

**S4.** HRMS Spectrum of Compound **3**

**
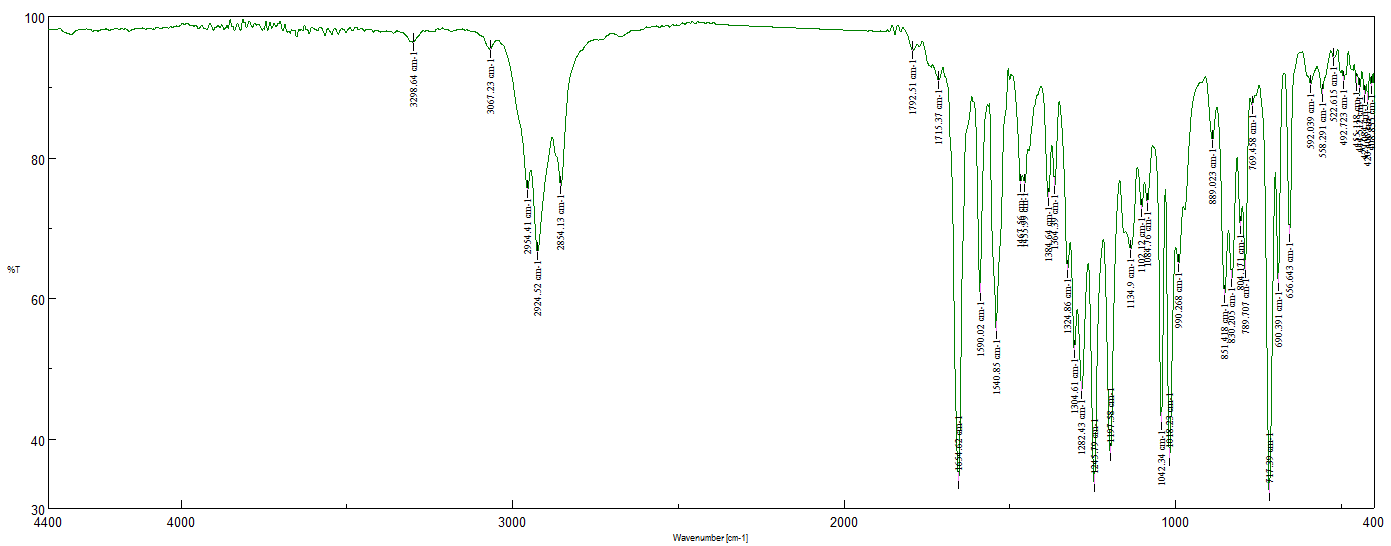
**

**S5.** FTIR Spectra of Compound **4**


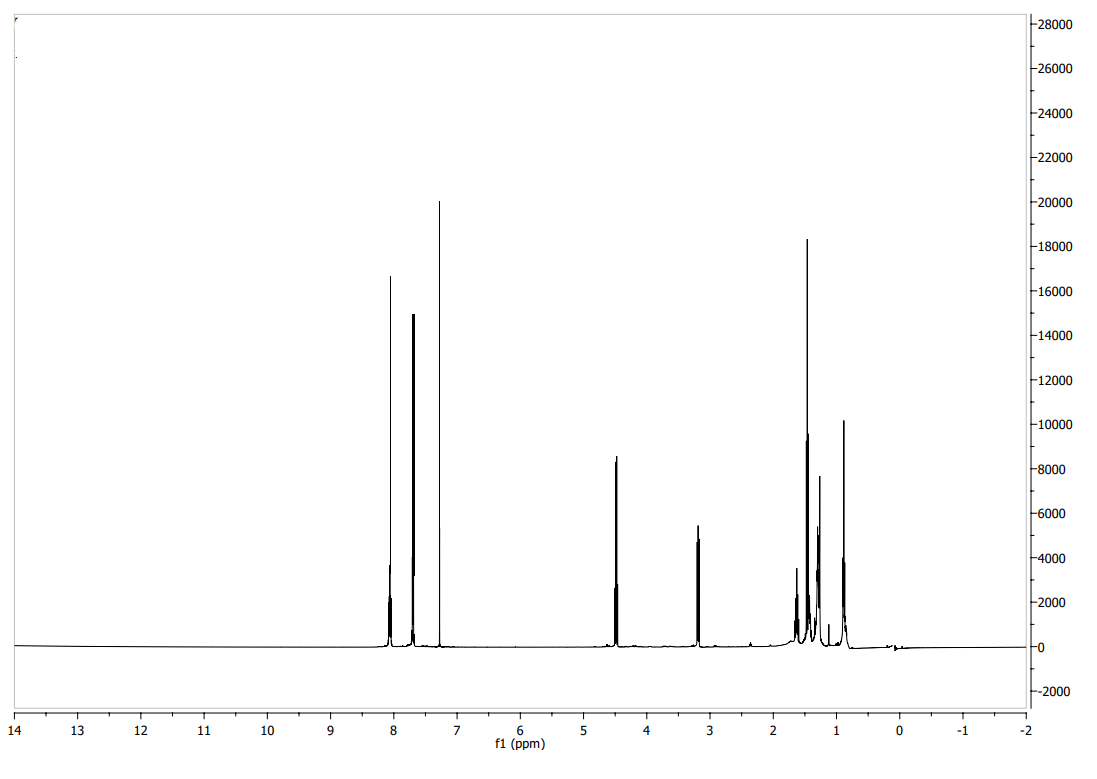


**S6**. ^1^H NMR Spectrum of Compound **4**


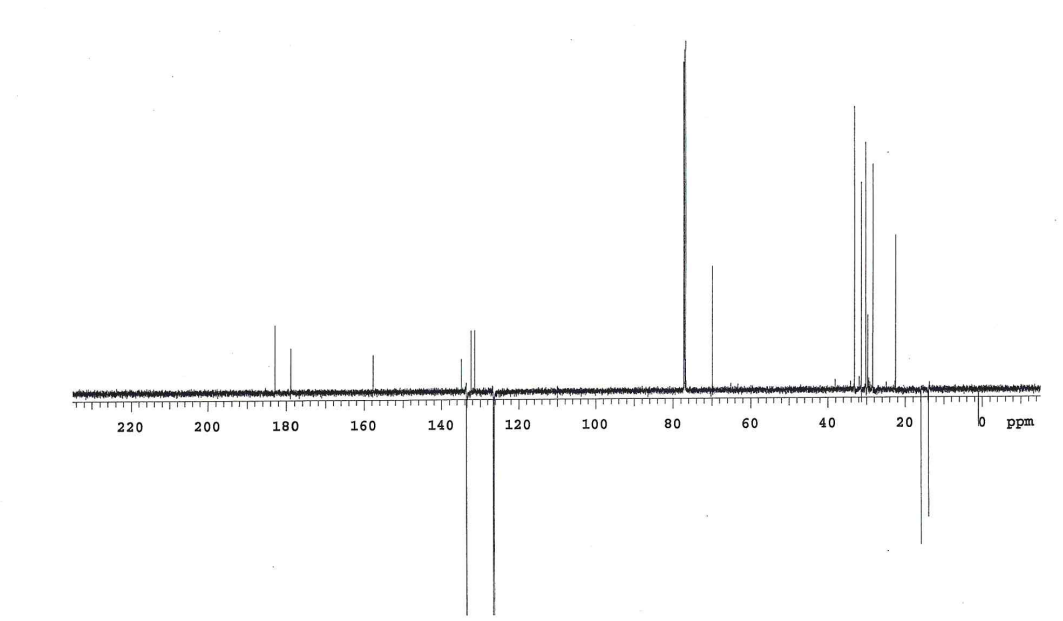


**S7.** ^13^C NMR(APT) Spectrum of Compound **4**

**
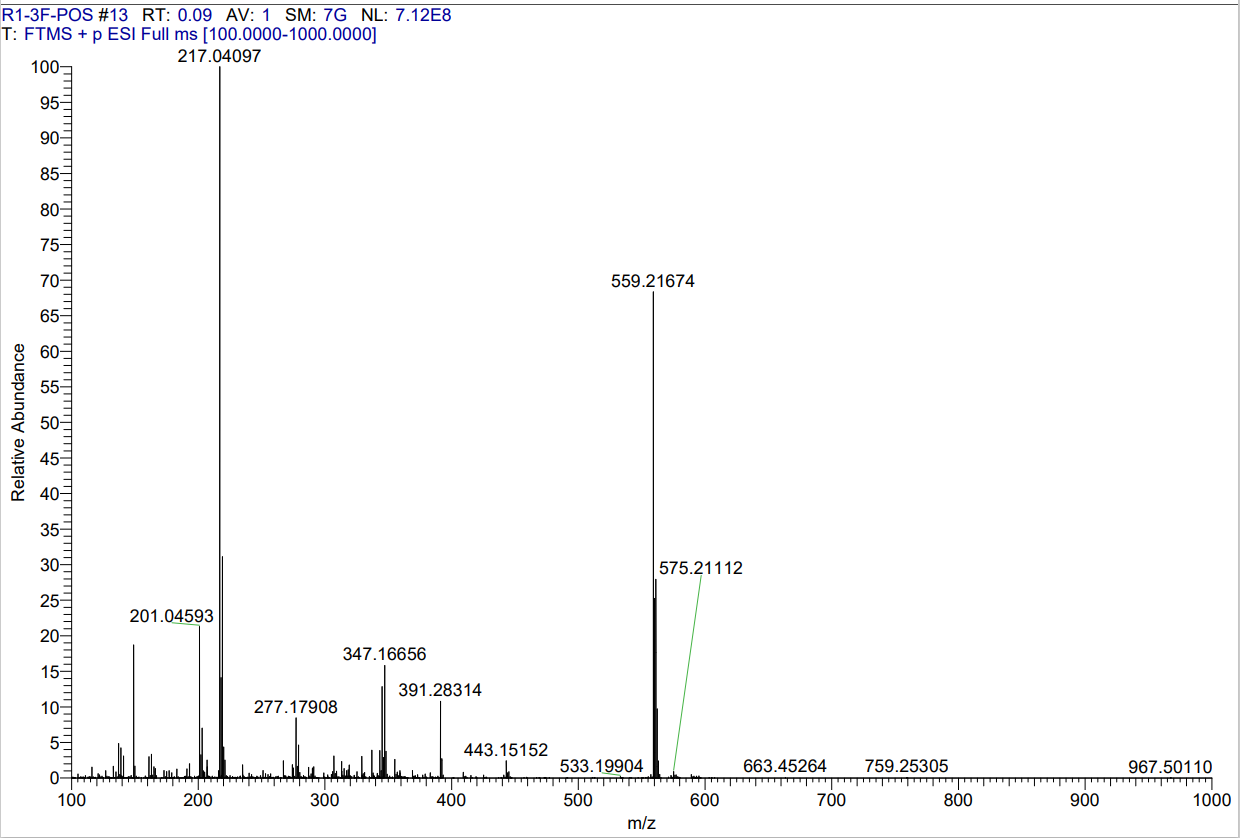
**

**S8.** HRMS Spectrum of Compound **4**

**
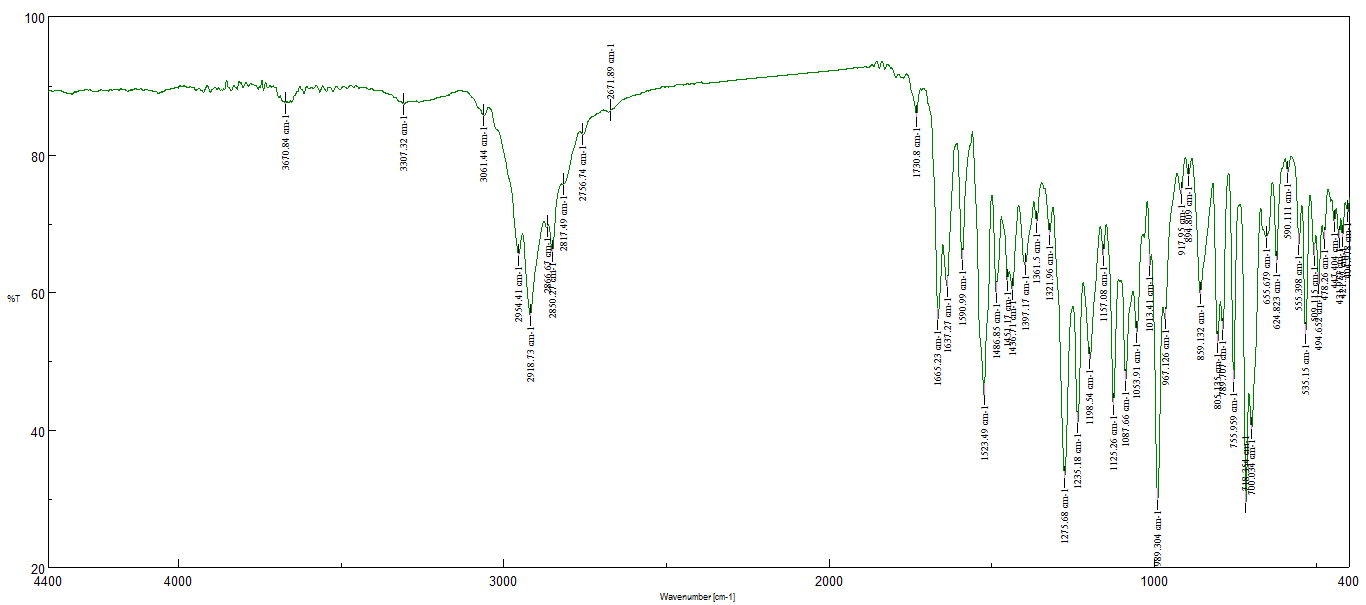
**

**S9.** FTIR Spectra of Compound **5**


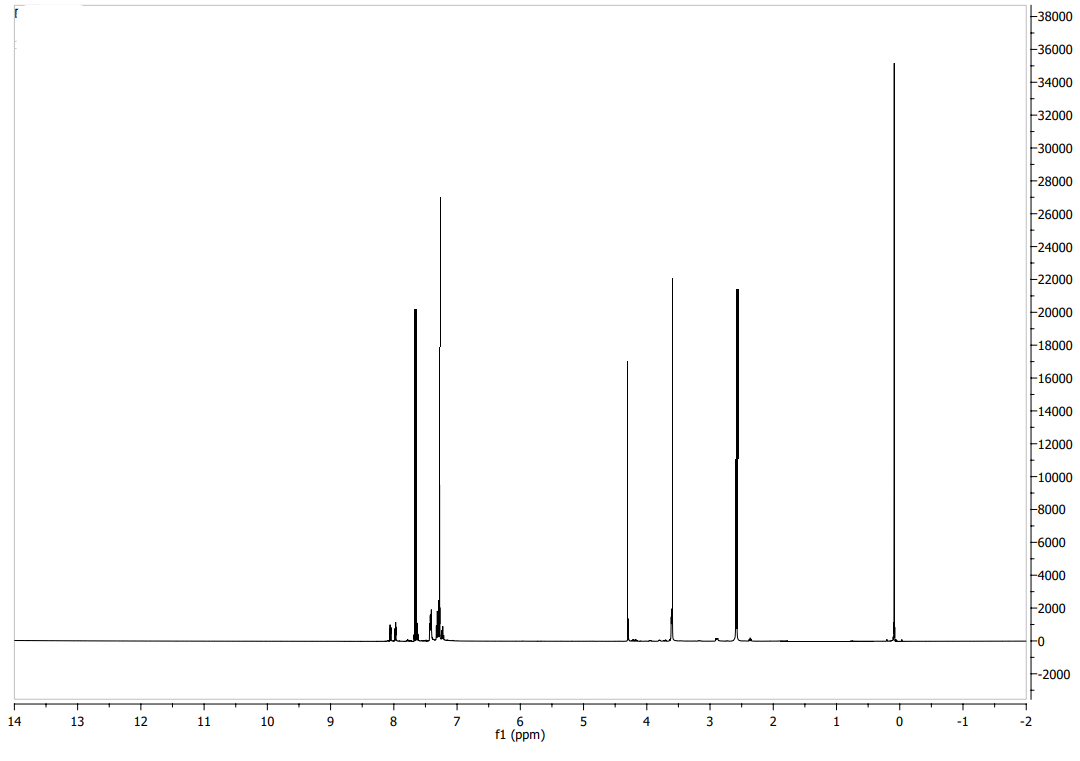


**S10**. ^1^H NMR Spectrum of Compound **5**


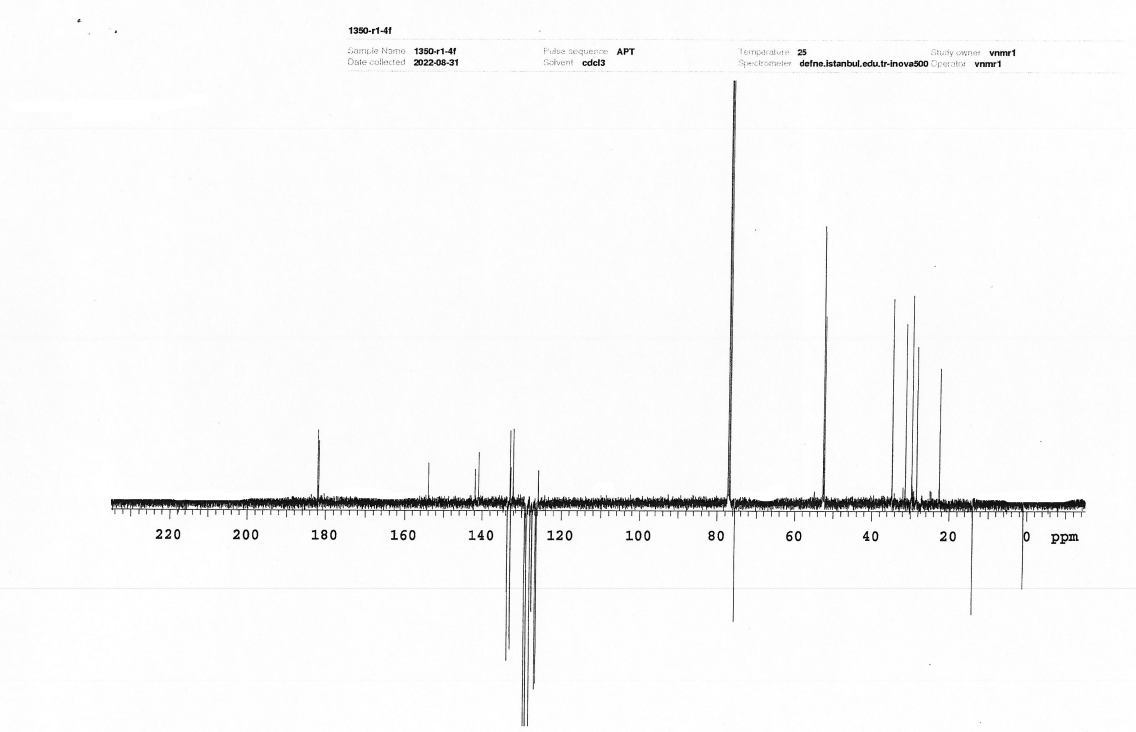


**S11.** ^13^C NMR(APT) Spectrum of Compound **5**


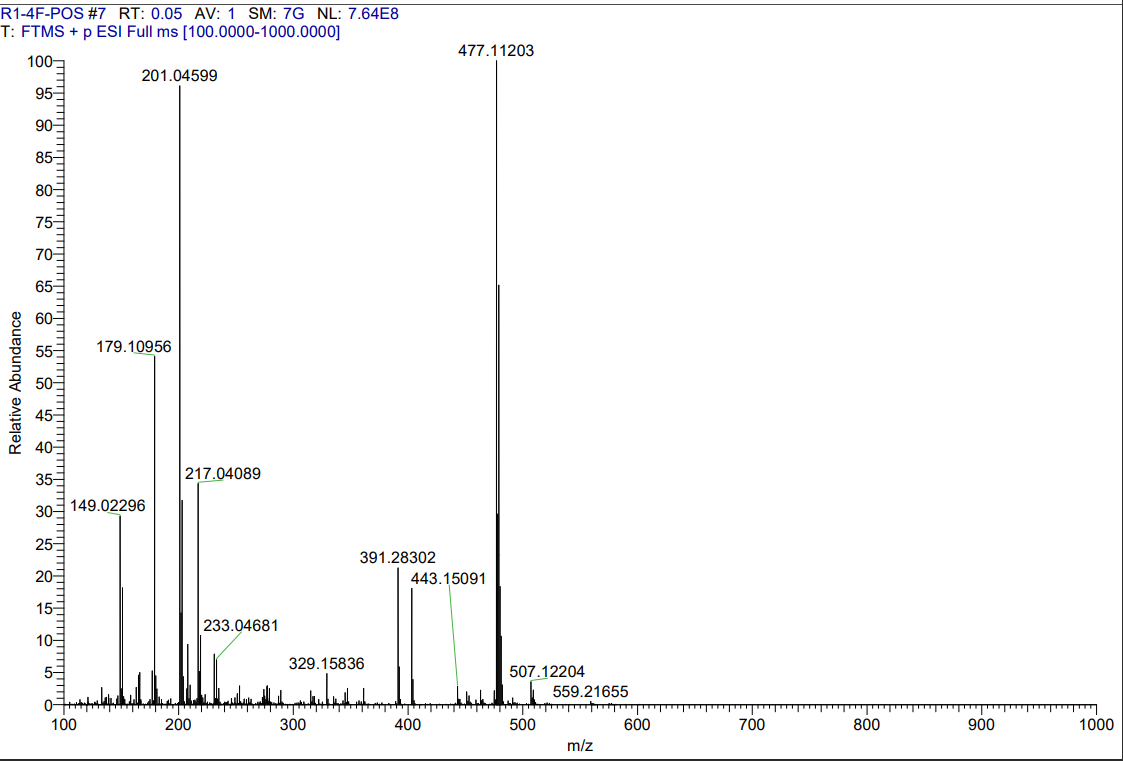


**S12.** HRMS Spectrum of Compound **5**

**
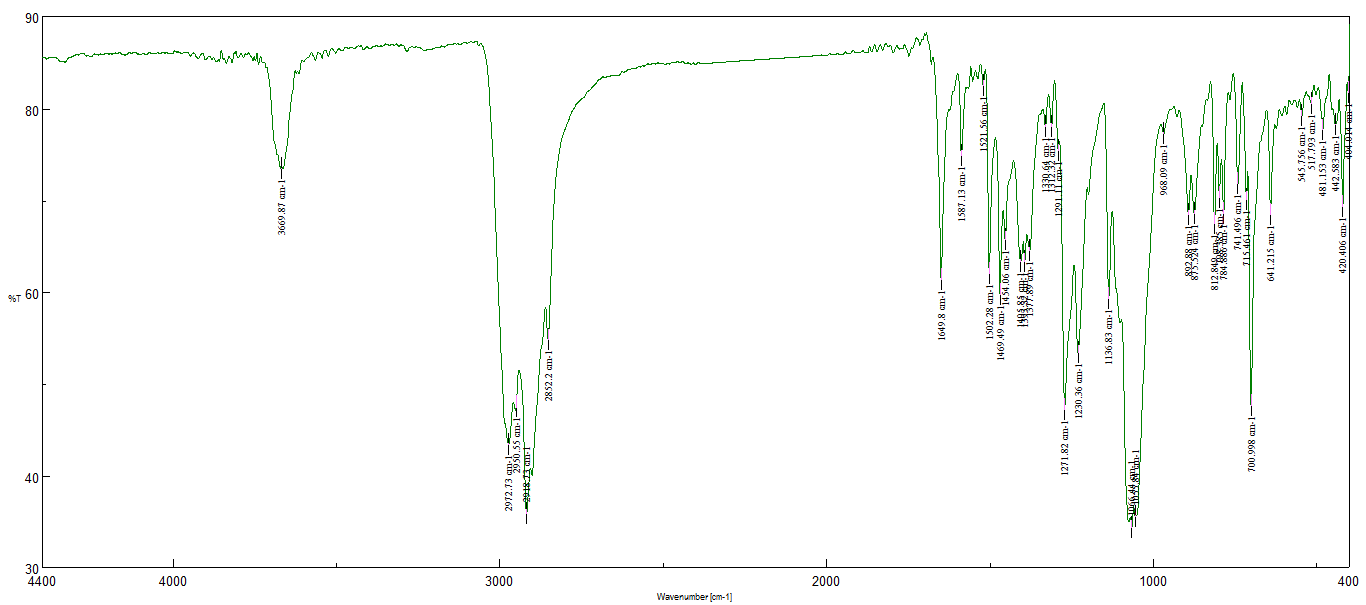
**

**S13.** FTIR Spectra of Compound **6**


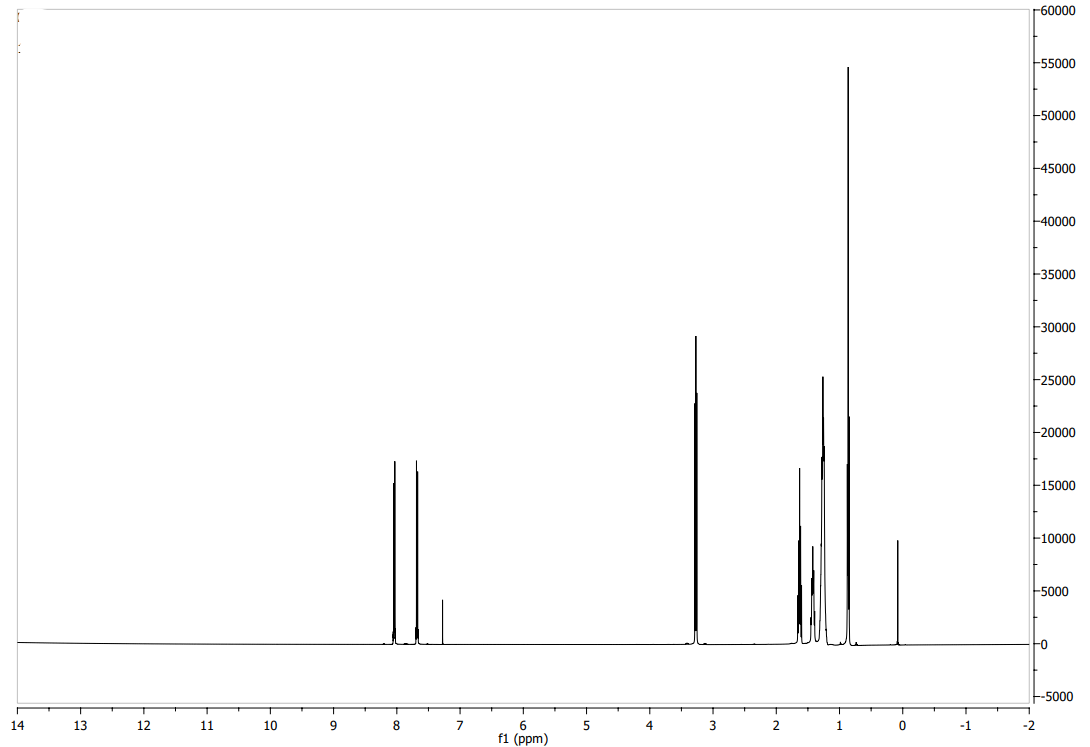


**S14**. ^1^H NMR Spectrum of Compound **6**


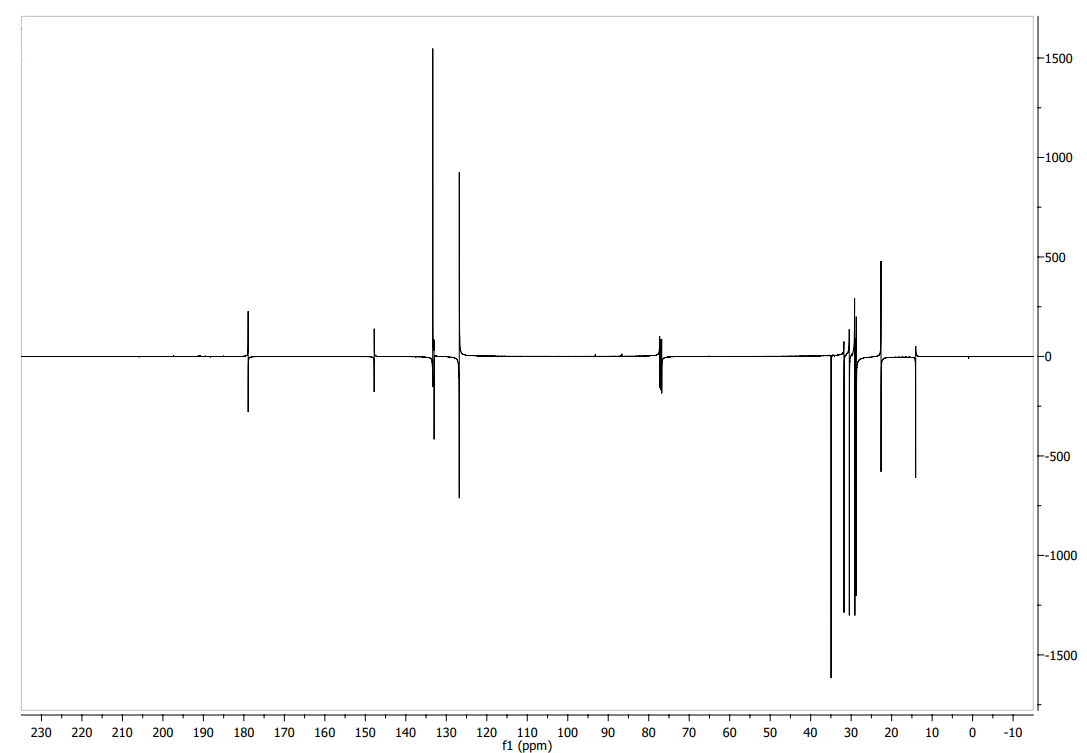


**S15.** ^13^C NMR(APT) Spectrum of Compound **6**

**
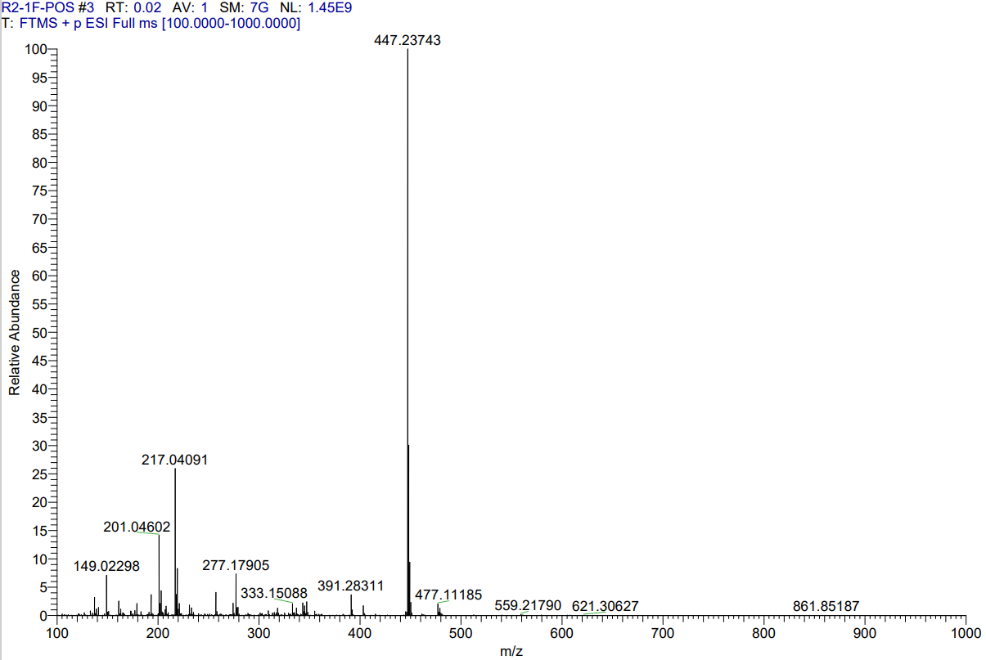
**

**S16.** HRMS Spectrum of Compound **6**

**
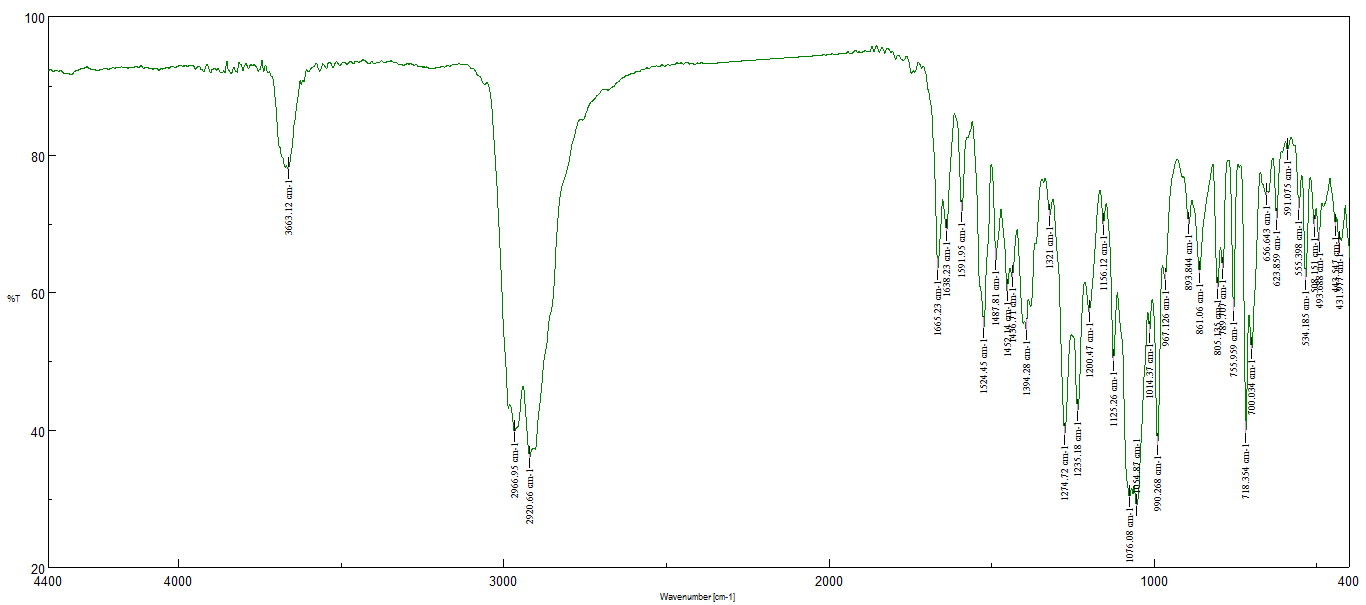
**

**S17.** FTIR Spectra of Compound **7**


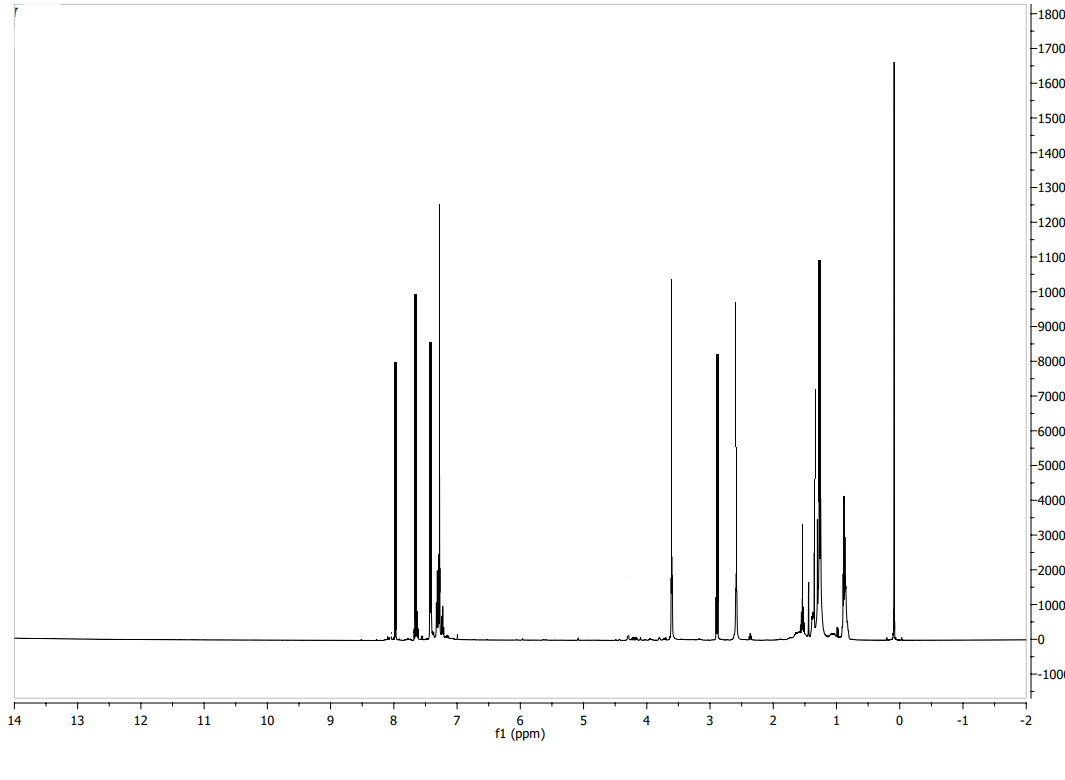


**S18**. ^1^H NMR Spectrum of Compound **7**


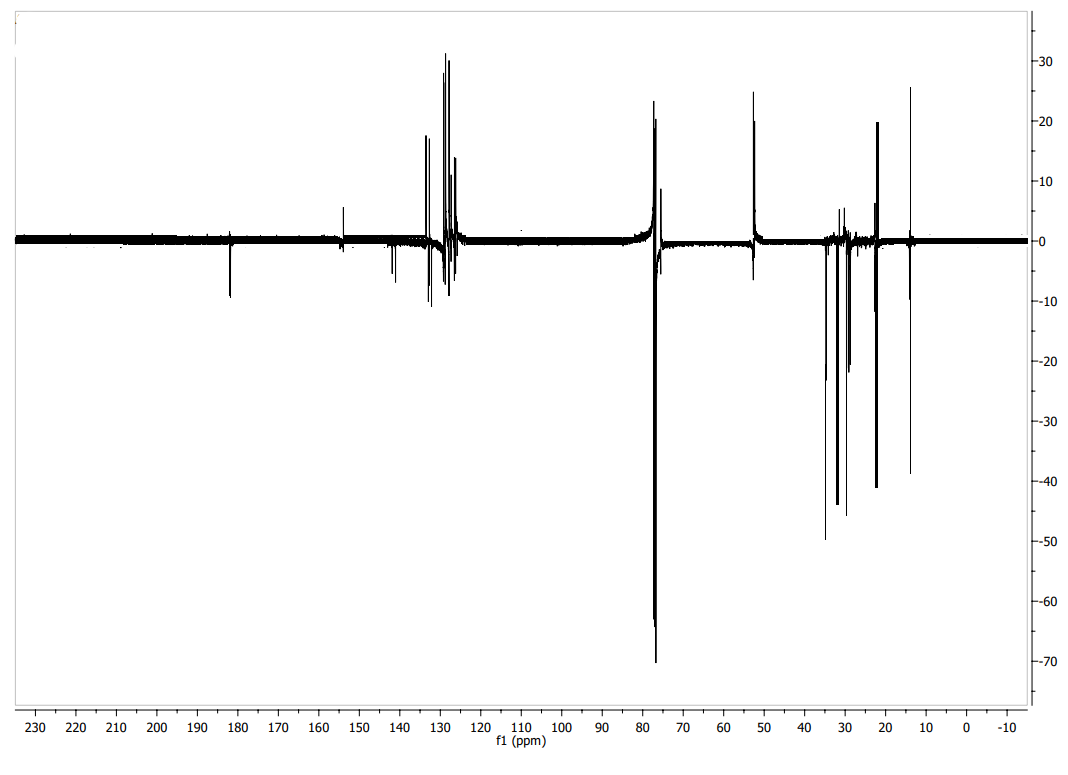


**S19.** ^13^C NMR(APT) Spectrum of Compound **7**

**
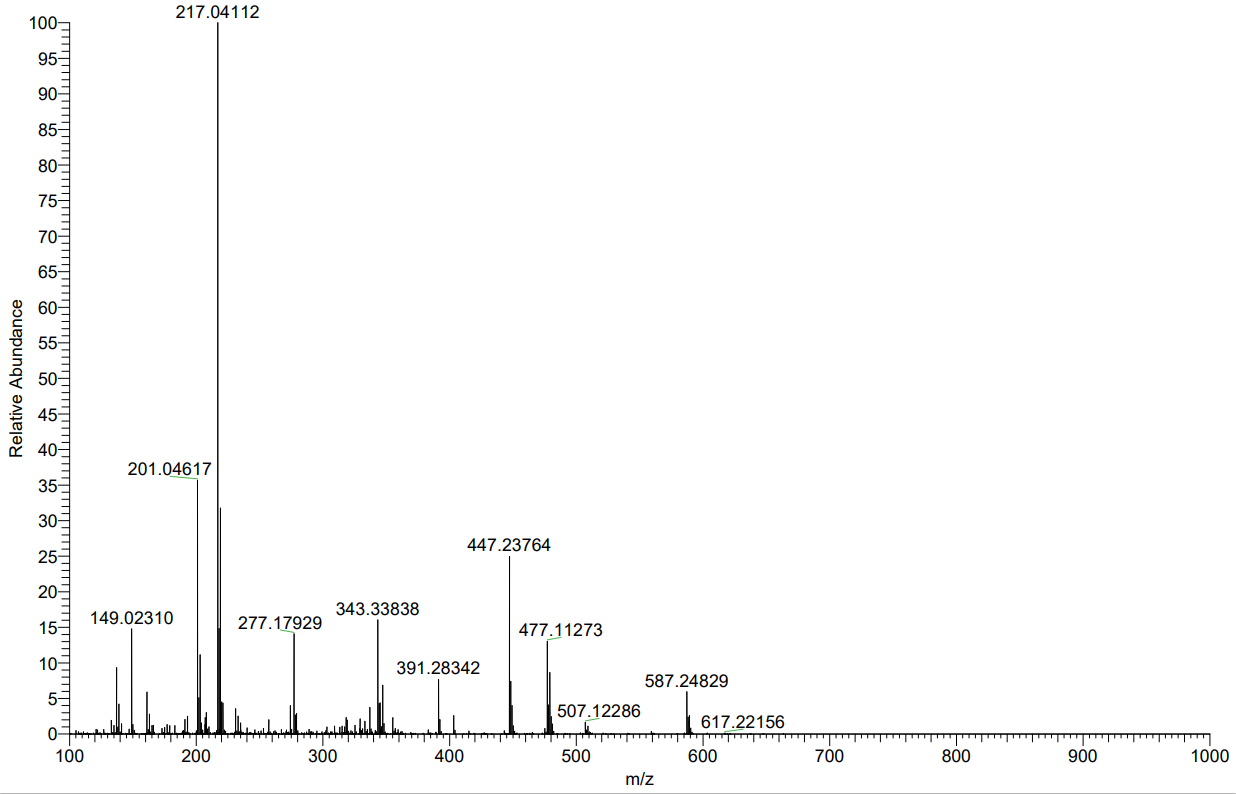
**

**S20.** HRMS Spectrum of Compound **7**

**
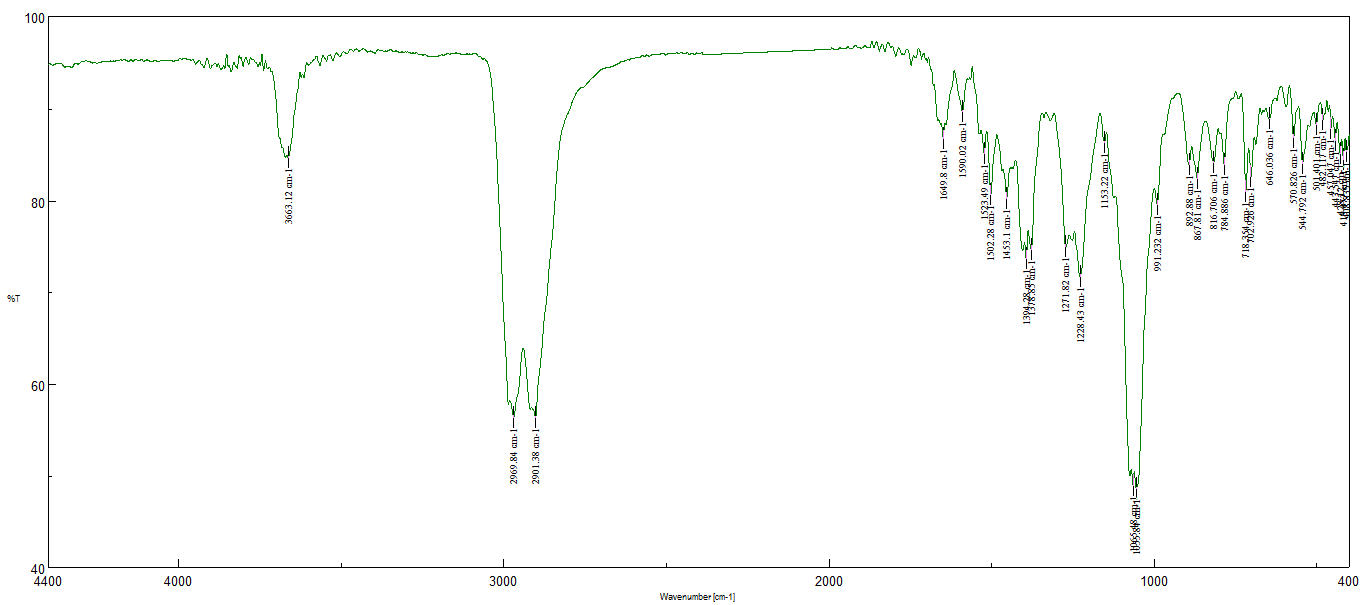
**

**S21.** FTIR Spectra of Compound **8**


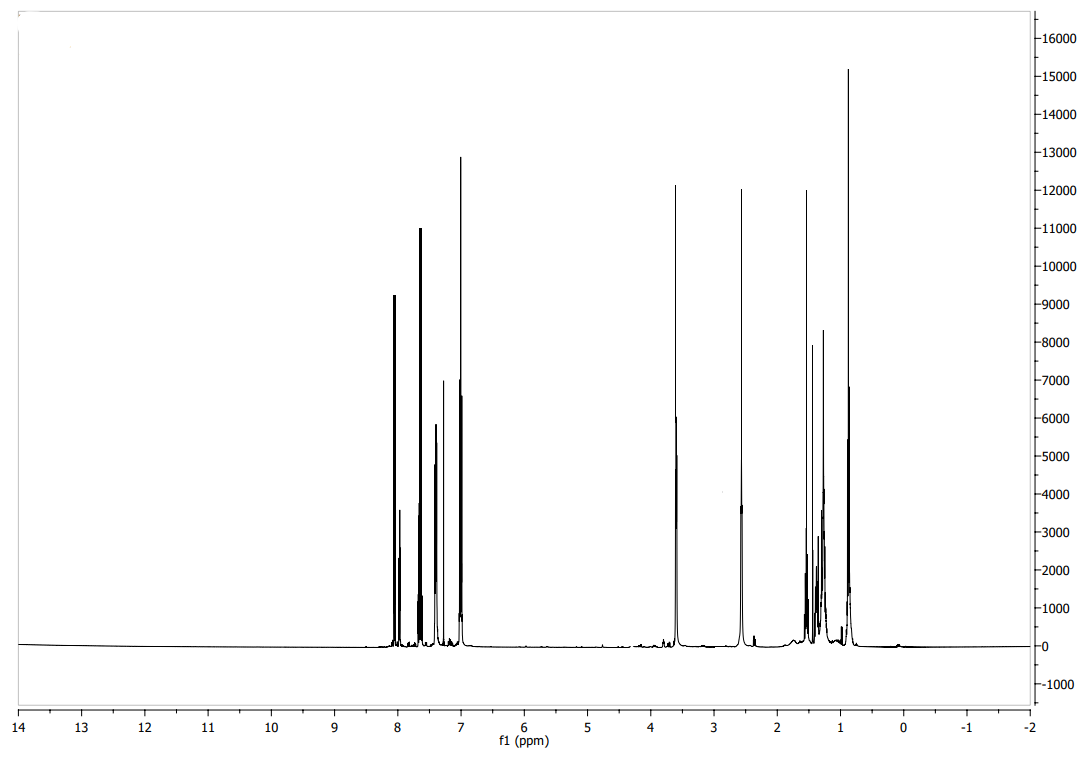


**S22.** ^1^H NMR Spectrum of Compound **8**


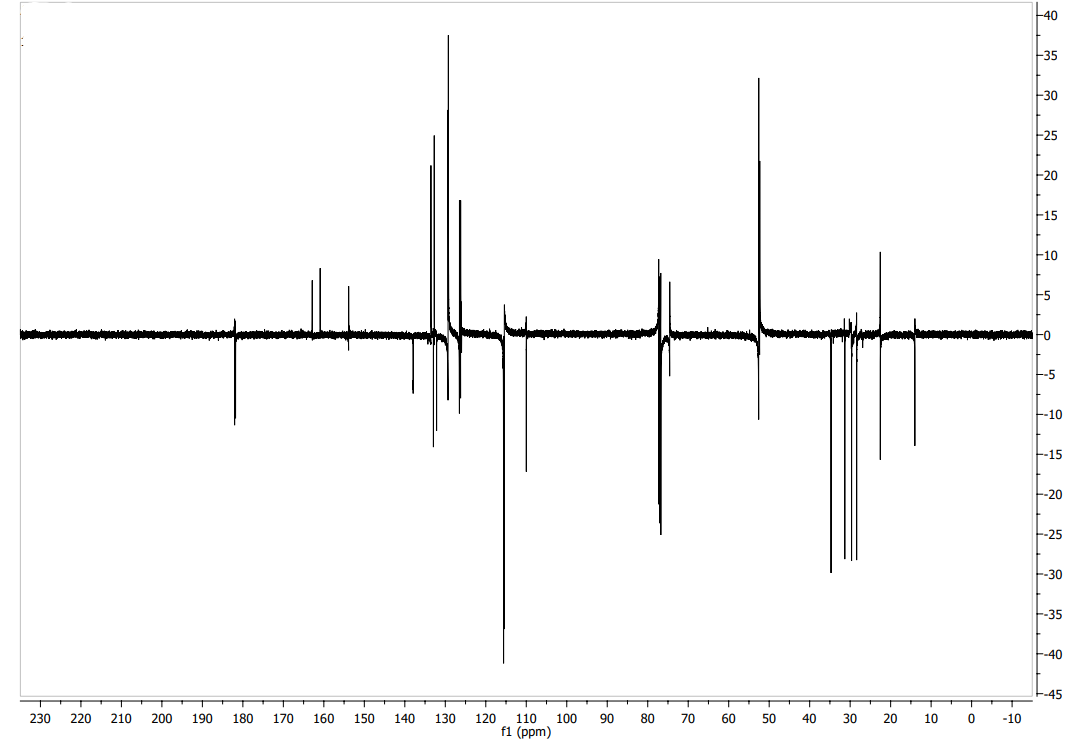


**S23.** ^13^C NMR(APT) Spectrum of Compound **8**

**
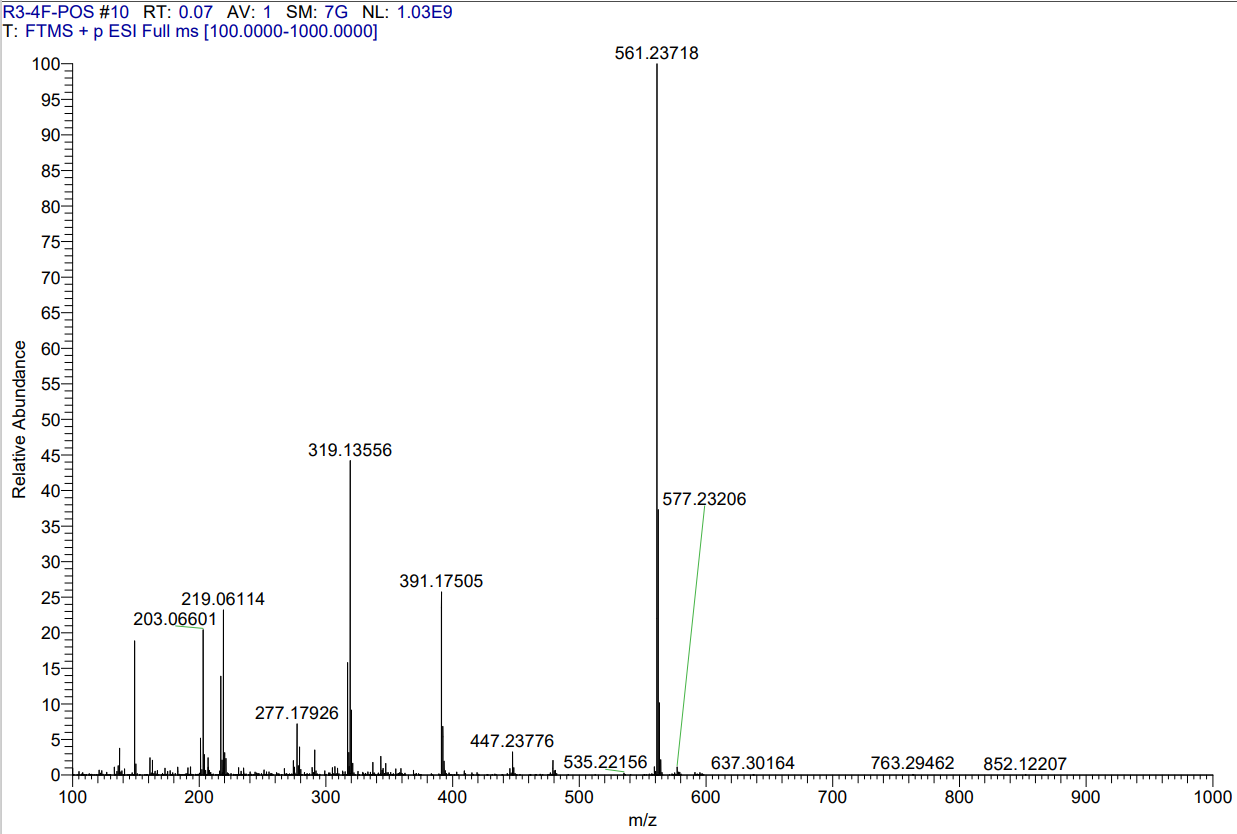
**

**S24.** HRMS Spectrum of Compound **8**

**
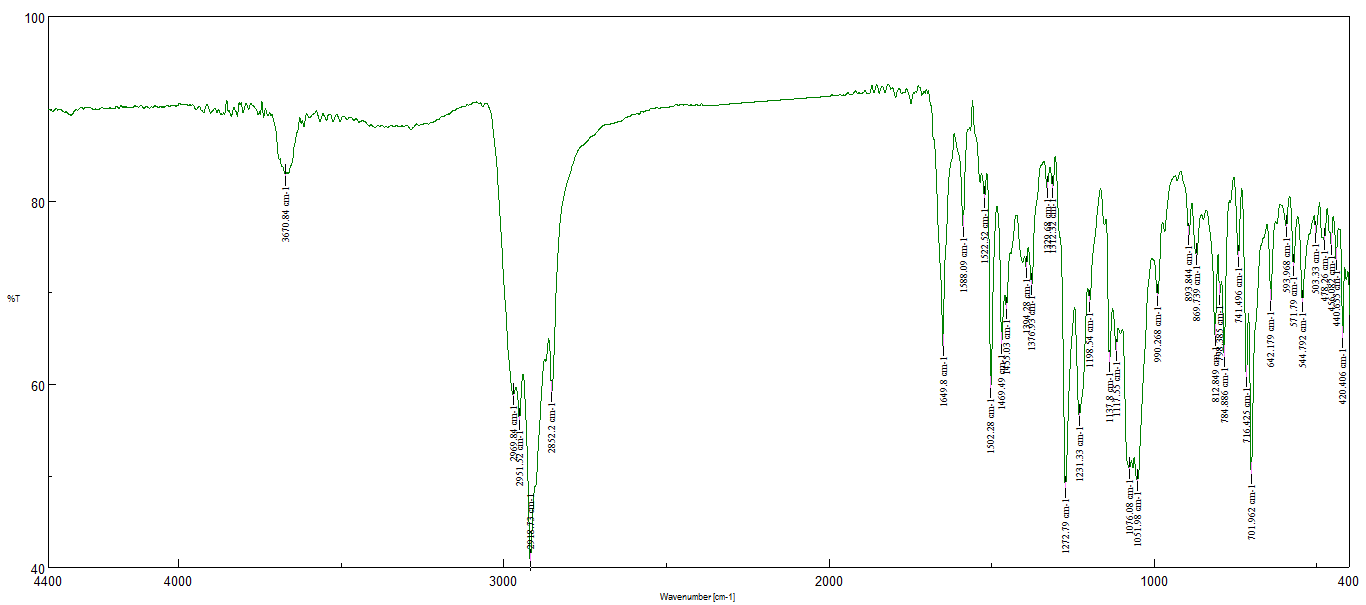
**

**S25.** FTIR Spectra of Compound **9**


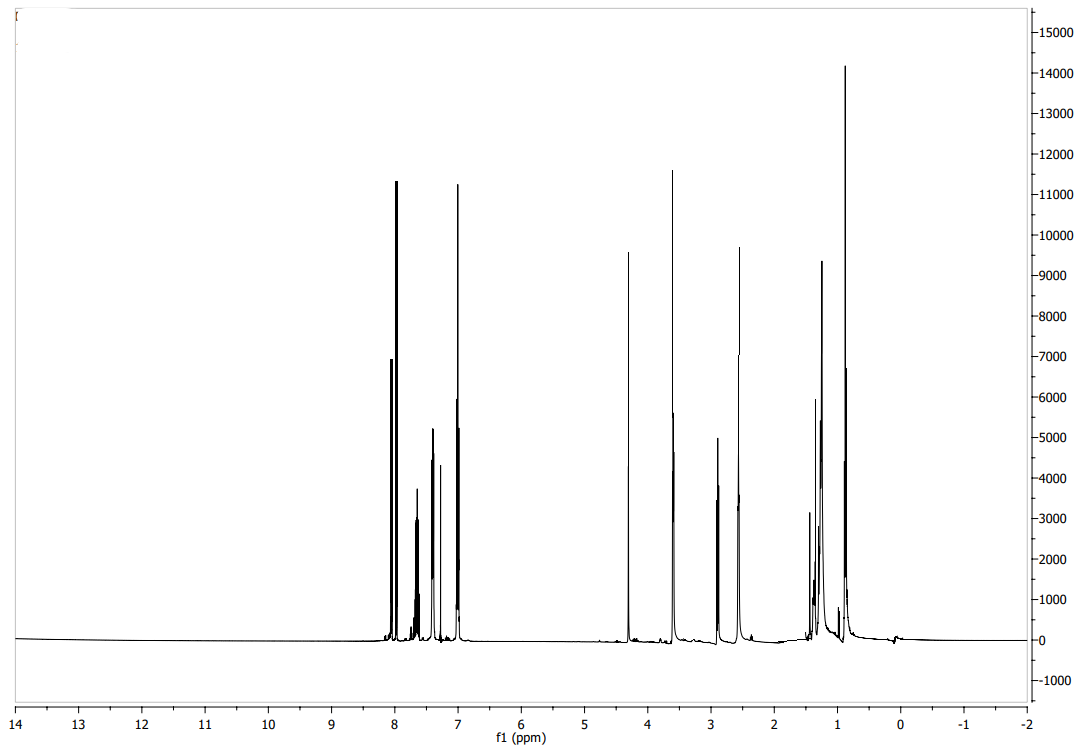


**S26.** ^1^H NMR Spectrum of Compound **9**


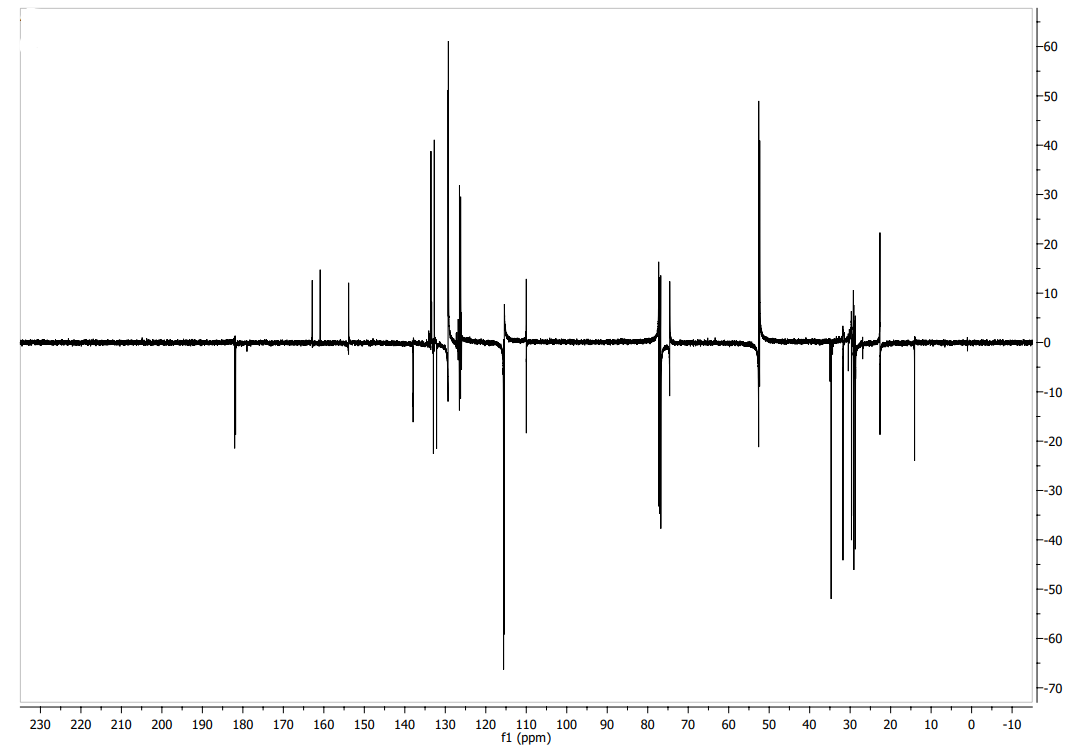


**S27.** ^13^C NMR(APT) Spectrum of Compound **9**

**
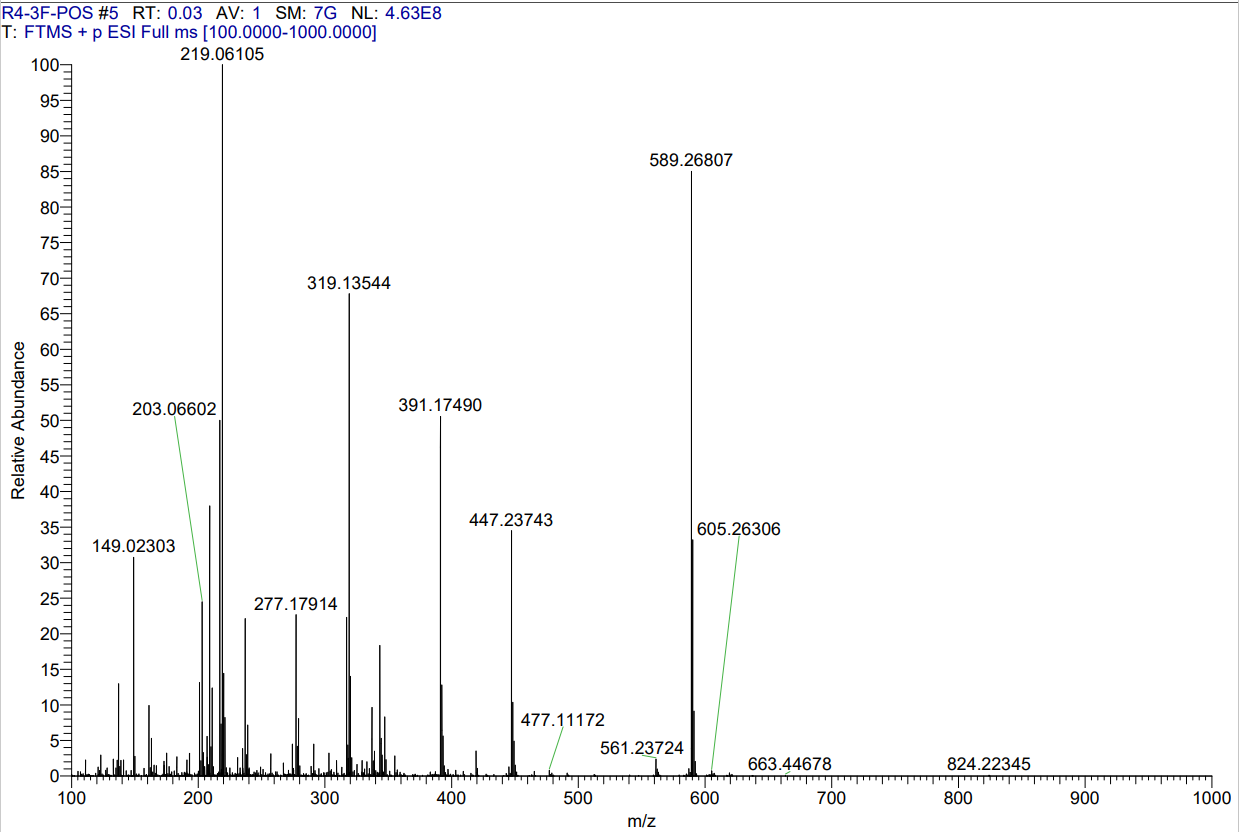
**

**S28.** HRMS Spectrum of Compound **9**

**
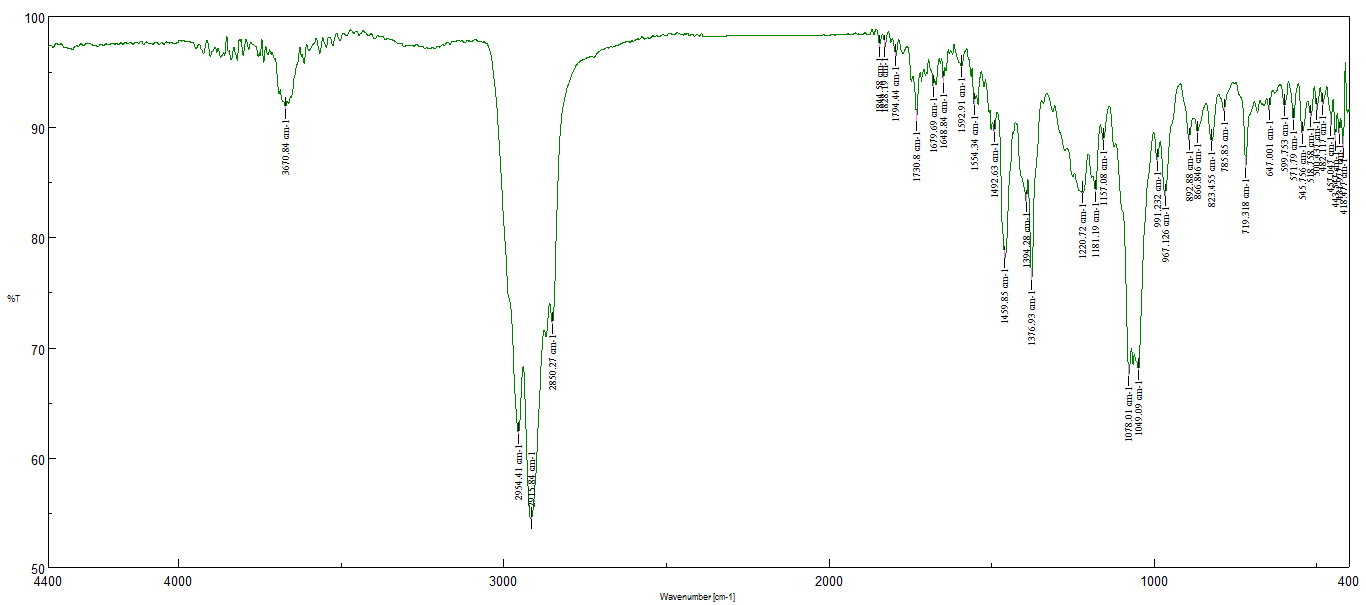
**

**S29.** FTIR Spectra of Compound **10**


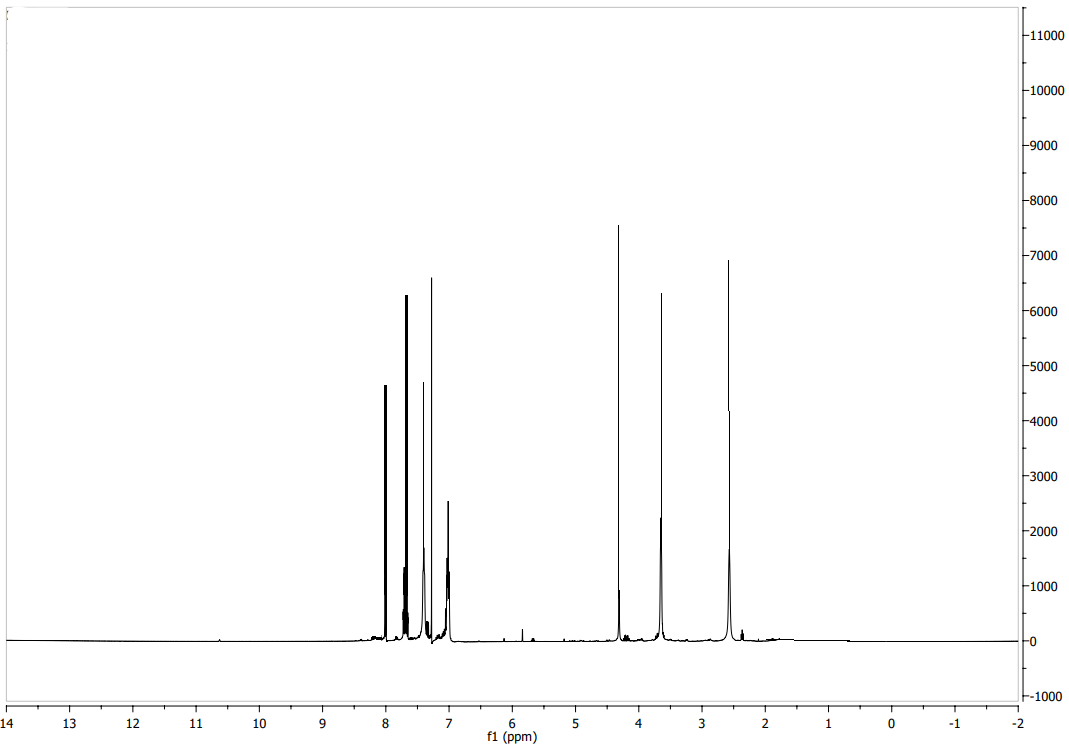


**S30.** ^1^H NMR Spectrum of Compound **10**


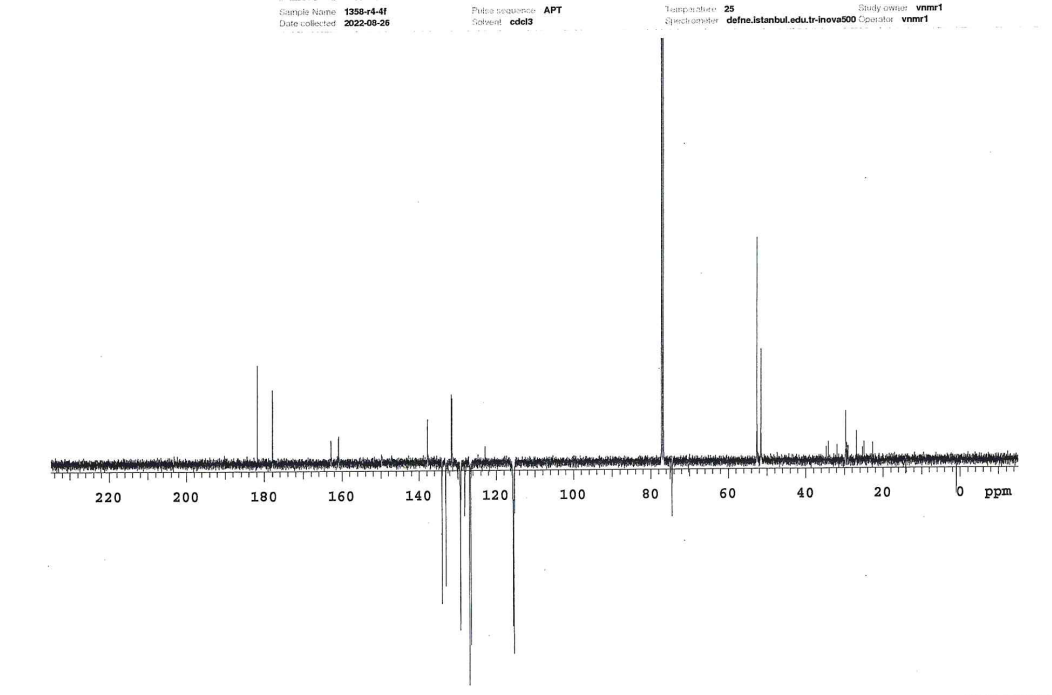


**S31.** ^13^C NMR(APT) Spectrum of Compound **10**

**
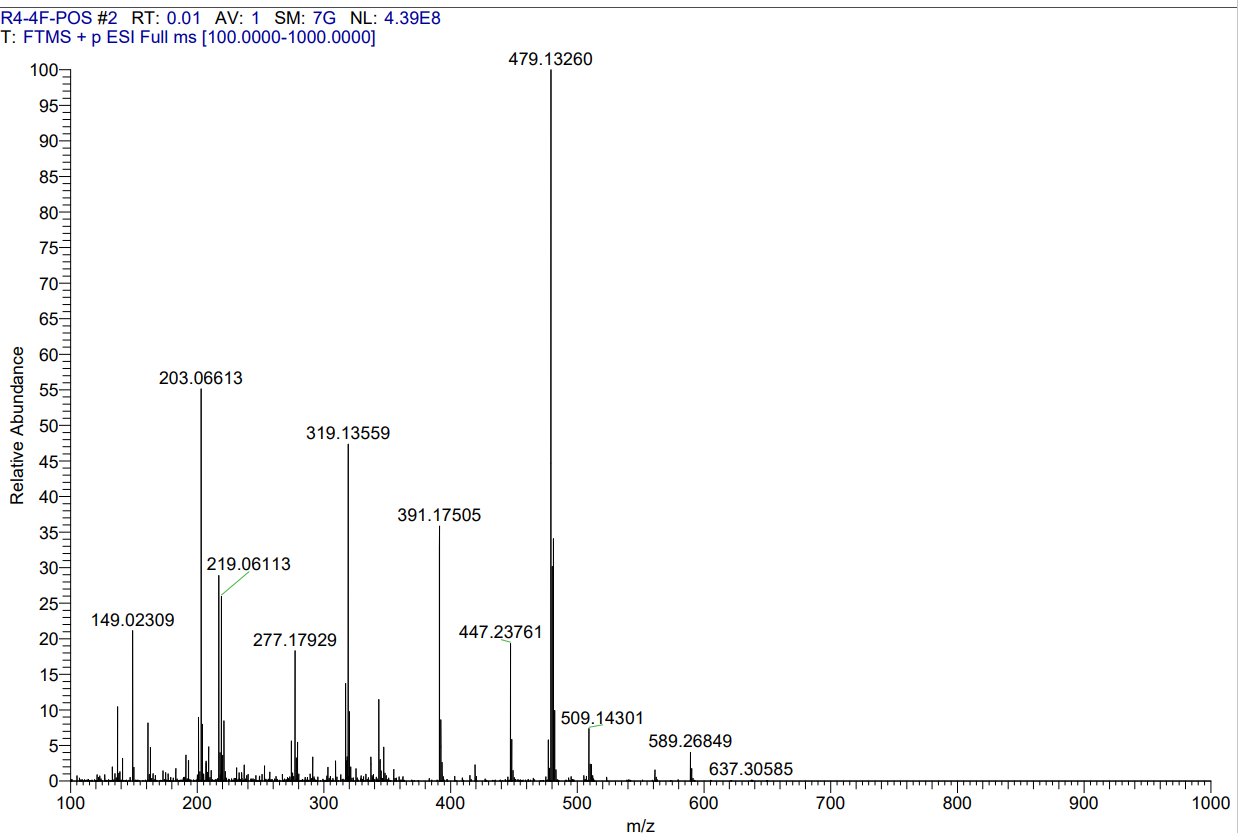
**

**S32.** HRMS Spectrum of Compound **10**
